# Molecular structure of the ESCRT-III-based archaeal CdvAB cell division machinery

**DOI:** 10.1073/pnas.2525941123

**Published:** 2026-01-16

**Authors:** Tina Drobnič, Ralf Salzer, Tim Nierhaus, Margaret Ke Xin Jiang, Dom Bellini, Astrid Steindorf, Sonja-Verena Albers, Buzz Baum, Jan Löwe

**Affiliations:** ^a^Medical Research Council Laboratory of Molecular Biology, Structural Studies Division, Cambridge CB2 0QH, United Kingdom; ^b^Molecular Biology of Archaea and Centre for Integrative Biological Signalling Studies, Faculty of Biology, University of Freiburg, Freiburg 79104, Germany

**Keywords:** archaea, cell division, cytokinesis, ESCRT-III, membrane remodelling

## Abstract

Most prokaryotes divide using filaments of the tubulin-like FtsZ protein, while some archaea employ instead ESCRT-III-like proteins and their filaments for cell division and cytokinesis. The alternative archaeal system comprises Cdv proteins and is thought to bear some resemblance to ESCRT-III-based membrane remodeling in other domains of life, including eukaryotes, especially during abscission. Here, we present biochemical, crystallographic, and cryo-EM studies of the *Sulfolobus* Cdv machinery. CdvA, an early non-ESCRT component, adopts a PRC‐domain/coiled-coil fold and polymerizes into long double-stranded helical filaments, mainly via hydrophobic interfaces. Monomeric CdvB adopts the canonical ESCRT-III fold in both a closed and a distinct “semiopen” conformation. Soluble CdvB2 filaments are composed of subunits in the closed state, appearing to transition to the open, active state only when polymerized on membranes. Short N-terminal amphipathic helices in all CdvB paralogues, B, B1, and B2, mediate membrane binding and are required for liposome recruitment in vitro. We provide a molecular overview of archaeal ESCRT-III-based cytokinesis machinery, the definitive demonstration that CdvB proteins are bona fide ESCRT-III homologues, and reveal the molecular basis for membrane engagement. Thus, we illuminate conserved principles of ESCRT-mediated membrane remodeling and extend them to an anciently diverged archaeal lineage.

## Significance

Membrane remodeling by ESCRT-III proteins is a fundamental and conserved process across the tree of life. The archaeal ESCRT-III-based cell division system (Cdv) drives cytokinesis in many archaeal groups, yet the molecular architecture of its components remained unknown, making it difficult to decipher the molecular mechanisms employed for cell division and cytokinesis in these organisms. We present structures of the Cdv machinery in *Sulfolobus* organisms that have been used previously to study the cell division process. We show that CdvA forms unexpected antiparallel helical filaments, while the ESCRT-III homologues retain the canonical fold they share with eukaryotic proteins. We demonstrate that N-terminal helices mediate membrane binding and that membrane contact, rather than polymerization alone, likely triggers activation of Cdv ESCRT-IIIs.

Cell division culminates in cytokinesis, the physical separation of one cell into two. This involves dramatic membrane remodeling in all cells. The underlying molecular machinery varies across life: bacteria and most archaea utilize FtsZ (tubulin)-based systems (1, 2), while eukaryotes use a contractile actomyosin ring and ESCRT-III (endosomal sorting complex required for transport III) proteins for cytokinesis (3, 4). In Crenarchaea, including S*ulfolobus*, cytokinesis is mediated by the ESCRT-III-like Cdv (cell division) system (5, 6).

**Fig. 1.**
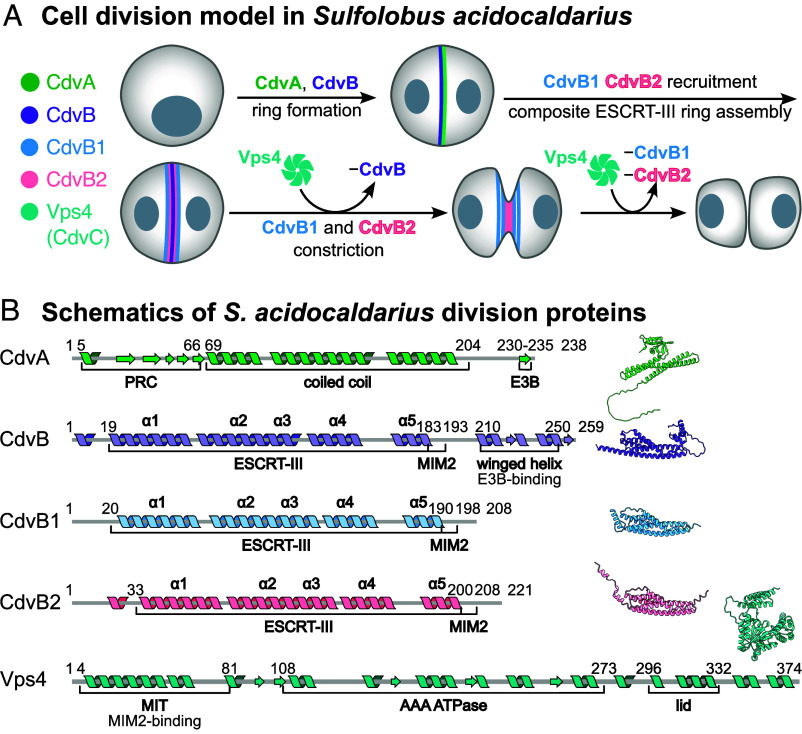
Archaeal Cdv cell division system. (*A*) Model of cell division in *S. acidocaldarius*. Adapted from refs. 11 and 12. (*B*) Secondary structure and domain organization of *Sulfolobus* Cdv cell division proteins. ESCRT-III helices α1-α5 are indicated. Residue numbers correspond to *S. acidocaldarius* sequences, secondary structure predicted with Jpred 4 (13). AlphaFold2 models (14) of *S. acidocaldarius* proteins are shown on the right.

The *Sulfolobus* Cdv system is composed of several division ring-forming components, CdvA, CdvB, CdvB1, and CdvB2, and a dedicated AAA+ ATPase CdvC (also called Vps4). It was first described in *Sulfolobus acidocaldarius*, with additional members identified in the related *Sulfolobus islandicus* (7). Division in *Sulfolobus* follows a defined sequence (Fig. 1*A*). CdvA and CdvB first form a ring at the midcell. It functions as a noncontractile scaffold to recruit CdvB1 and CdvB2, forming a composite division ring. CdvB is then actively removed from the ring by Vps4 and degraded by the proteasome. This allows CdvB1 and CdvB2 to remodel the membrane, driving constriction. The two proteins show a spatially patterned distribution – CdvB1 is localized more peripherally, while CdvB2 is at the constricting membrane neck. There, it is believed to perform the final membrane abscission step, as evidenced by its strong loss-of-function phenotype (5, 8–10).

CdvA is unique to the archaeal Cdv system (15). It is predicted to contain a PRC domain, a coiled-coil region, and a C-terminal ESCRT-III-binding (E3B) sequence. Though it lacks homology to any ESCRT, it templates downstream ESCRT-III assembly (12), directly binding CdvB via the E3B peptide (8).

Though lacking upstream homologs, the Cdv system is related to eukaryotic ESCRT-IIIs. Canonical ESCRT-III proteins share a five-helix core (α1 to α5) and remodel membranes by polymerization, transitioning from a “closed” monomeric (16, 17) to an “open” polymeric form (18–20). CdvB, CdvB1, and CdvB2 are believed to be ESCRT-III homologues. All are predicted to share the core ESCRT-III domain and a MIM2 motif for interaction with Vps4 (Fig. 1*B*). The three paralogues likely arose from two gene duplications from an ancestral CdvB (21).

Despite the central role of the Cdv system in division, high-resolution structures are available only for CdvC (22, 23). Thus, the molecular mechanisms underlying Cdv function remain largely unclear. Here, we present molecular structures of CdvA, CdvB, and CdvB2. We show that CdvA adopts a distinct fold and forms antiparallel helical filaments via hydrophobic interactions, while monomeric CdvB and polymerized CdvB2 show canonical ESCRT-III folds. Unexpectedly, soluble CdvB2 filament subunits remain in the closed state and appear to transition to the open conformation only when polymerized on a membrane. Membrane association is mediated by short N-terminal amphipathic α-helices in all CdvB paralogues. These findings reveal conserved principles of membrane remodeling across domains of life.

## Results

### Constitutive Helical Filaments of CdvA Are Stabilized Via Hydrophobic Interactions.

**Fig. 2.**
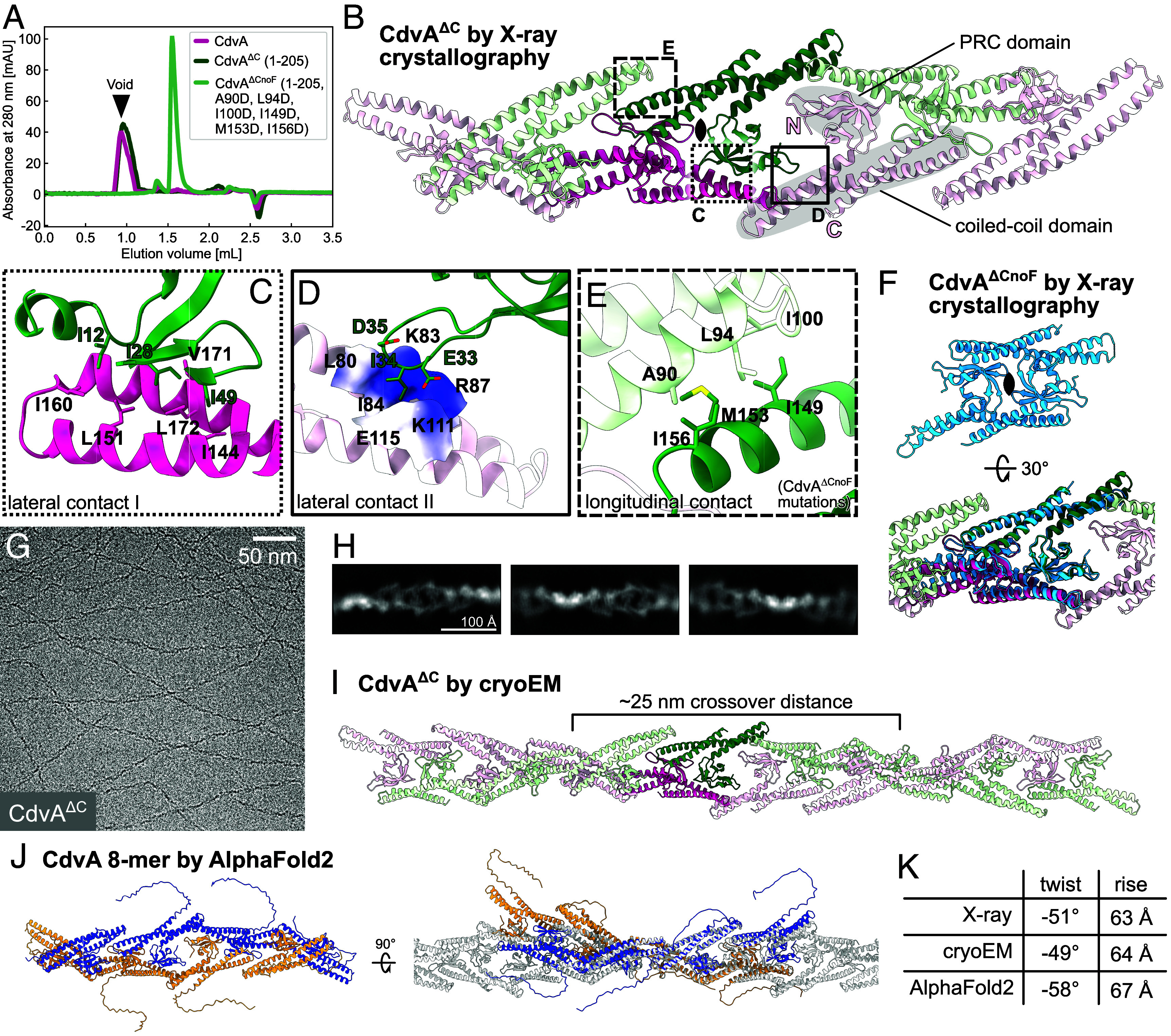
CdvA forms constitutive double-stranded helical filaments. (*A*) Size exclusion chromatography of CdvA constructs. Filament formation is abolished by mutating six hydrophobic residues (CdvA^∆CnoF^). (*B*) *S. islandicus* CdvA^∆C^ as determined by X-ray crystallography forms a dimer (darker color), which repeats to form a double-stranded helical filament in the protein crystal; strands (protofilaments) shown in green and pink. Each subunit contains a PRC domain and a three-helix coiled-coil domain. (*C*) Hydrophobic and (*D*) hydrophilic lateral contacts between CdvA^∆C^ protofilaments. View is rotated by 180**°** relative to panel *B*. (*E*) Longitudinal hydrophobic contacts between coiled-coils within a protofilament. Mutating these six residues prevents filament formation (panel *A*). (*F*) Nonpolymerizing mutant (CdvA^∆CnoF^) forms a dimer similar to CdvA^∆C^ as determined by X-ray crystallography. Structures overlaid in the *Bottom* panel. (*G*) Cryoelectron micrograph and (*H*) 2D class averages of CdvA^∆C^ filaments. (*I*) Cryo-EM structure of CdvA^∆C^ matches X-ray filament structure. (*J*) AlphaFold2 prediction of eight CdvA chains agrees with experimental CdvA^∆C^ filament structures. *Right* panel: overlay on cryo-EM structure (gray). (*K*) Table of approximate helical parameters of the crystal, cryo-EM, and AlphaFold2 models. For crystal and AlphaFold2 models, parameters were calculated using relion_helix_toolbox on maps simulated with ChimeraX *molmap*.

CdvA is unique to archaeal ESCRT-III systems. It is the first Cdv component recruited to the midcell in *Sulfolobus*, where it templates assembly of subsequent ESCRT-III rings (12). CdvA from *Metallosphaera sedula* has been shown to form double-stranded filaments that were reportedly stabilized by DNA (15). To investigate how it polymerizes, we purified full-length *S. islandicus* CdvA for structural analysis. It formed filaments that only dissolved in 1M CHES buffer at pH 11. To aid in crystallization, we removed the flexible C-terminal linker and E3B peptide region (CdvA^∆C^, residues 1–205). The truncation did not disrupt polymerization (Fig. 2*A*) and it enabled us to determine the molecular structure of crystallized CdvA^∆C^ at 2.9 Å resolution. Interestingly, CdvA^∆C^ formed an antiparallel double-stranded helical filament in the crystal (Fig. 2*B*), with a dimer as the repeating unit (dark green and magenta chains).

Inspecting the filament structure revealed that lateral interactions between strands involve a PRC domain on one strand contacting the coiled-coil domain of two subunits on the opposite strand. Lateral contact I is mediated by extensive hydrophobic interactions (Fig. 2*C*), while lateral contact II consists of three hydrophobic residues nested in an otherwise electrostatic pocket (Fig. 2*D*). Within each protofilament (strand), the coiled-coil domains contact one another in a head-to-tail manner. This interface is made up of six hydrophobic residues, three on each subunit (Fig. 2*E*). To test the role of this longitudinal interaction on CdvA’s ability to polymerize, we mutated the six positions to aspartates (A90D, L94D, I100D, I149D, M153D, I156D). This resulted in a nonpolymerizing CdvA^∆CnoF^ protein (Fig. 2*A*) without altering the protein fold, as confirmed by a 2.2 Å crystal structure of CdvA^∆CnoF^ (Fig. 2*F*). The crystallized dimer closely matched the dimeric repeat unit of the CdvA^∆C^ filament, but did not assemble a filament itself. Aligning the dimers with PyMOL’s (v2.5) align command yielded a backbone RMSD of 1.56 Å after outlier rejection, reflecting conservation of the overall fold. Taken together, this indicates that the hydrophobic coiled-coil interface is essential for CdvA filament formation.

To verify the CdvA filament observed in the protein crystals we turned to cryo-EM (Fig. 2 *G* and *H*). The final helical reconstruction at 4.1 Å resolution (Fig. 2*I*) was very similar to the filament observed in the crystals, having the same subunit arrangement and almost identical helical parameters. A similar polymer assembly was also recapitulated by AlphaFold2 predictions (Fig. 2*J*), albeit with somewhat different calculated helical parameters (Fig. 2*K*). However, the model retained the same fold, subunit organization, longitudinal and lateral interfaces as the experimental structures. Taken together, these data confirm that CdvA polymerizes into double-stranded, antiparallel, helical filaments with strong hydrophobic interaction surfaces.

### Archaeal CdvB Has an ESCRT-III Fold.

**Fig. 3.**
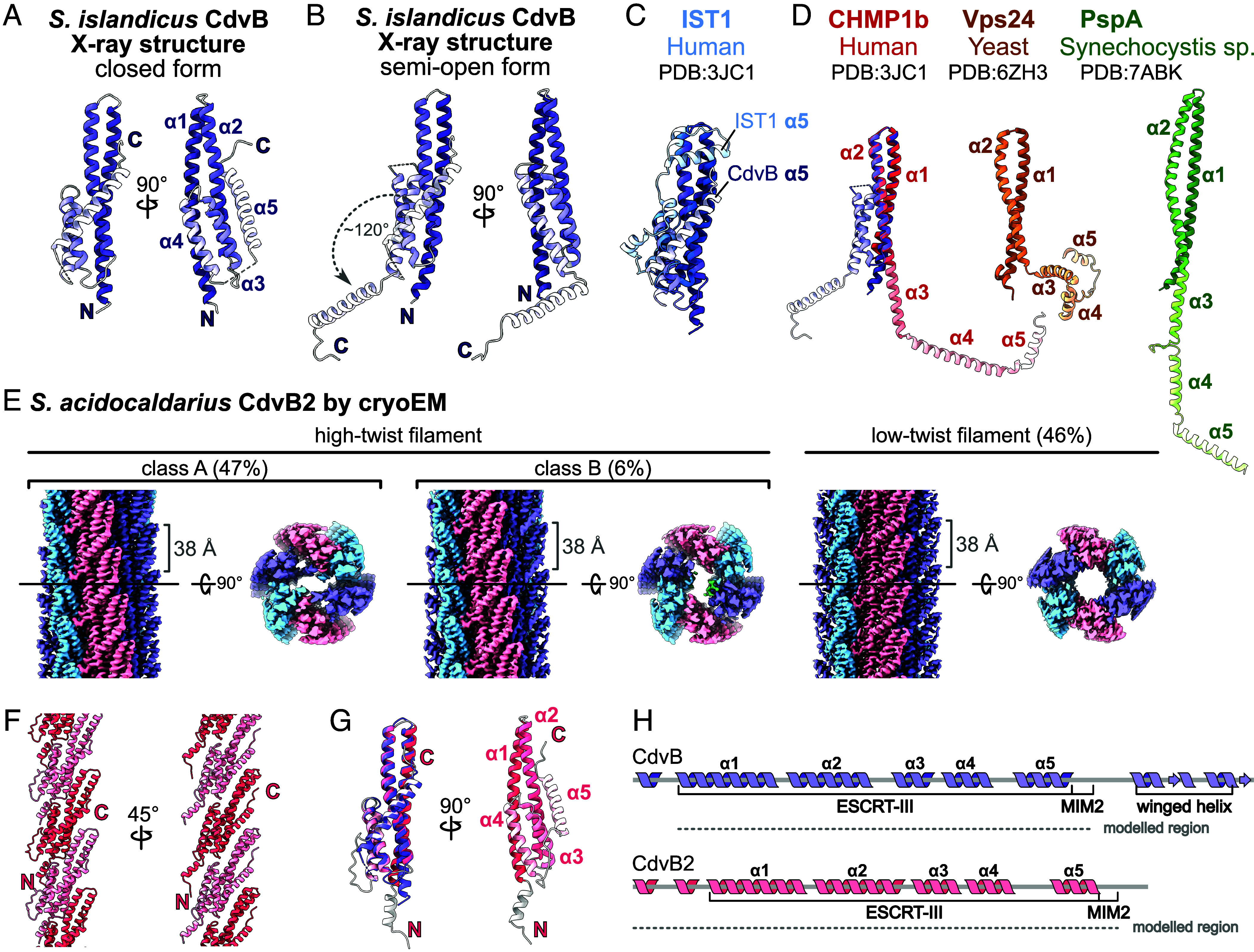
Structures of archaeal CdvB and CdvB2 ESCRT-IIIs. (*A*) *S. islandicus* CdvB in closed conformation as determined by X-ray crystallography (P 4_1_ 2_1_ 2). Helices α1–α5 are colored in graded shades. (*B*) Alternate CdvB crystal form (P 2_1_ 2_1_ 2_1_) shows a semiopen conformation, where helix α5 swings open by ~120°; α5 closed position shown transparent. (*C*) Comparison of closed-form CdvB and human IST. (*D*) Comparison of semiopen CdvB with different open-form ESCRT-IIIs (CHMP1b, Vps24, and PspA). (*E*) *S. acidocaldarius* CdvB2 forms helical filaments of six protofilaments; showing side views and central slices along the XY axis. Three filament conformations fall into two main classes, high-twist and low-twist. Within a protofilament, intersubunit spacing (~38 Å) is consistent across classes. (*F*) Atomic model of a low-twist protofilament. N termini face the lumen, C termini are surface-exposed. (*G*) Atomic model of a low-twist CdvB2 monomer in closed form. *Left* panel: aligned to *S. islandicus* CdvB from panel A (purple). RMSD 1.13 Å after outlier rejection, aligned with ChimeraX matchmaker. (*H*) Cartoon schematics of CdvB and CdvB2, updated to reflect experimentally determined secondary structures.

We next focused on CdvB, the first ESCRT-III to be recruited to the site of division in Sulfolobales. The *S. islandicus* homologue was truncated to remove the C-terminal linker and winged helix domain (residues 193–259) for crystallization, and two methionine substitutions (I69M, I125M) enabled selenomethionine phasing. A structure of CdvB was determined at 2.6 Å (P 4_1_ 2_1_ 2), revealing the canonical ESCRT-III five-helix fold in the closed conformation (Fig. 3*A*). This is consistent with previously reported structures showing ESCRT-IIIs adopt a closed conformation as monomers (17, 18). A second CdvB crystal form (P 2_1_ 2_1_ 2_1_) revealed an alternate conformation at 2.2 Å resolution (Fig. 3*B*). Here, helices α1-α4 remain largely unchanged, but helix α5 is swung open approximately 120°, making it no longer folded over the α1-α2 hairpin. We term this conformation “semiopen” as only helix α5 changes its relative position while the rest remain as in the closed conformation.

We compared our CdvB structures with ESCRT-IIIs from organisms in other domains of life. Superposition of the closed-form CdvB and human IST1 highlights the conserved ESCRT-III fold between archaea and eukaryotes (Fig. 3*C*). They differ mainly in the position of helix α5, which sits lower on the α1–2 hairpin in CdvB. Comparing the semiopen CdvB structure with a range of different ESCRT-III structures reveals that CdvB can adopt a distinct conformation (Fig. 3*D*). In open-form ESCRT-IIIs from bacteria to humans, helices α3–5 unfurl with α3 becoming a continuous extension of α2. In semiopen CdvB, helices α3–4 remain packed to the side of the α1–2 hairpin. This differs from the “extended” conformation seen in yeast Vps24 filaments (24) (Fig. 3*D*, ochre), where helices α3–5 move away from the α1–2 hairpin, but are not fully extended.

### A Closed-Conformation CdvB2 Filament Buries Its Amphipathic Helices.

ESCRT-III monomers can form polymers that bind to and remodel membranes. To further investigate archaeal Cdv polymerization, we sought to structurally characterize CdvB polymers. Polymerization could be induced by the presence of either CHAPS or CHAPSO, or lipid monolayers. However, the samples were not amenable to high-resolution cryo-EM due to bundling and a lack of helical twist, leading to preferred orientation (*SI Appendix*, Fig. S2 *A*–*C*).

We instead turned to CdvB2, which is thought to perform the final membrane abscission during cytokinesis, in the better-characterized *S. acidocaldarius* system (10). Adjusting the buffer pH to six triggered the formation of CdvB2 filaments, which were then imaged by cryo-EM (*SI Appendix*, Fig. S3*A*).

We solved the structure of *S. acidocaldarius* CdvB2 filaments in three different forms (Fig. 3*E*). 2D class averages revealed two main filament types, high-twist and low-twist (*SI Appendix*, Fig. S3*B*). The high-twist filaments were further separated into two classes (A and B). All three filament types consisted of six protofilaments with longitudinal subunit spacings of ~38 Å and differed slightly in their helical parameters. The primary difference between high-twist classes A and B was in their lumenal densities. Atomic modeling revealed that, in all filament forms, the N termini face the lumen of the filaments, while the C termini face outward. This is consistent with the C-terminal MIM2 motif being solvent-exposed and accessible for remodeling by Vps4. In all cases, we saw density for only the first few amino acids of the MIM2 motif, indicating flexibility.

As in the case of CdvB, CdvB2 also adopts the characteristic 5-helix ESCRT-III fold. Moreover, the structures of individual CdvB2 and CdvB chains are strikingly similar, despite different source organisms and oligomeric states [matchmaker in ChimeraX (v1.7) reported RMSD of 1.13 Å after outlier rejection, Fig. 3 *G*, *Left* panel]. However, in all three filament forms, the CdvB2 subunits were found in a closed conformation (Fig. 3 *F* and *G*), rather than in an open conformation as would be expected for a polymerized ESCRT-III protein. While truncated IST1 has been reported to generate polymers in a closed form as part of a composite polymer, to our knowledge this is the first example of an ESCRT-III assembling into a closed-form homopolymer.

**Fig. 4.**
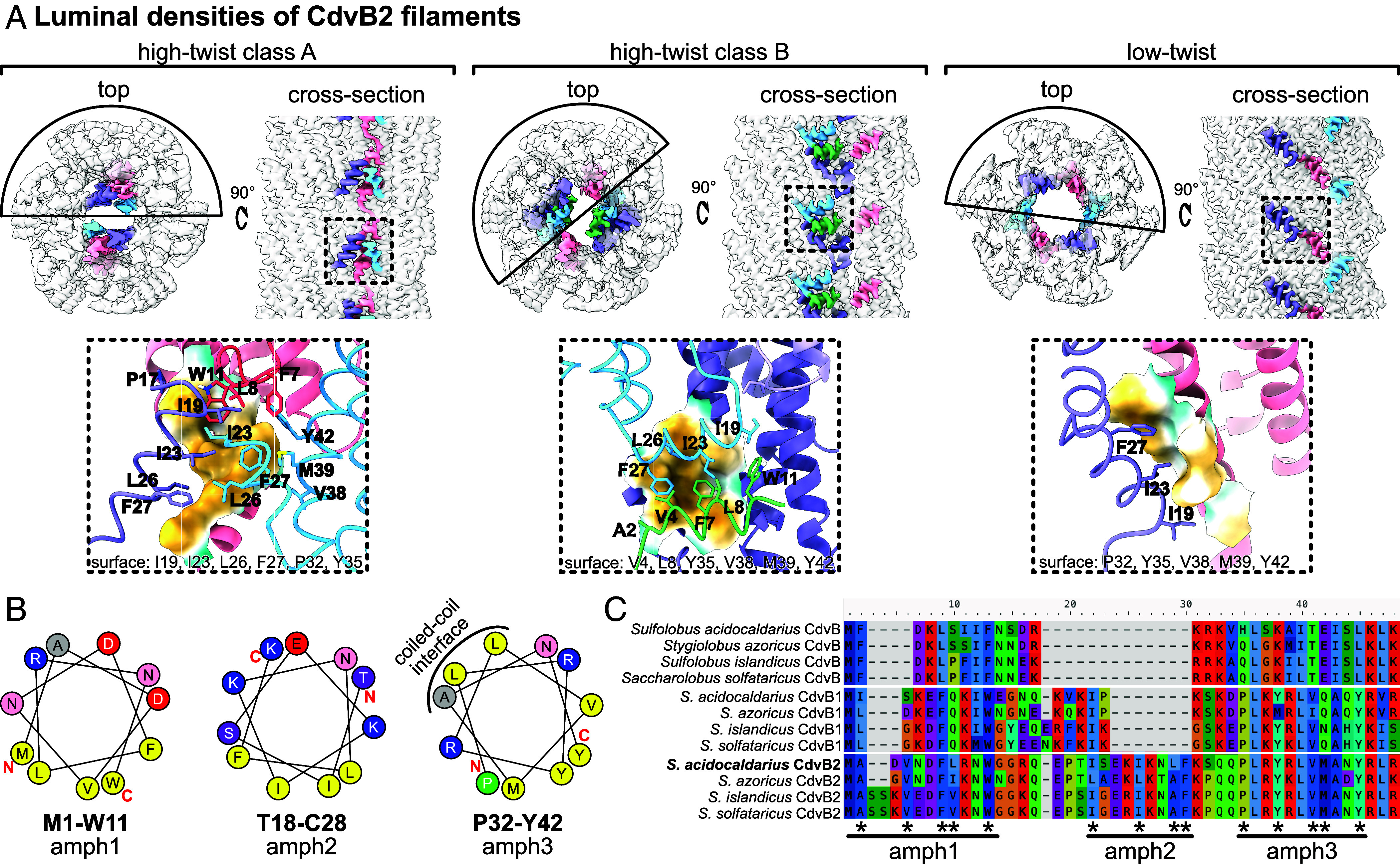
CdvB2 N-terminal region has amphipathic helices. (*A*) *Top* row: cryo-EM maps of CdvB2 showing different N-terminal arrangements (in color). *Bottom* row: atomic models of CdvB2 N termini, corresponding to regions in dashed boxes above. (*B*) Helical wheel diagrams of regions involved in hydrophobic interactions in panel *A*, showing they are short amphipathic helices. Calculated with the HeliQuest server (25). (*C*) Sequence alignment of N-terminal regions of CdvB, CdvB1, and CdvB2 from related *Sulfolobales*. Hydrophobic residues from panels *A* and *B* are highlighted with asterisks. Aligned with MAFFT online in auto mode (26).

One of the main differences that distinguishes the monomer structure from the three types of CdvB2 filament is the N-terminal region, which adopts different conformations (Fig. 4*A*). In all filament cases, hydrophobic residues consistently pack together and are shielded from solvent in the lumen. The hydrophobic residues tucked away on the inside of the filaments clustered in three regions, which were short α-helices with a hydrophobic face. The first two regions (M1-W11 and I19-F27) are short amphipathic α-helices (named here amph1 and amph2), while the third (P32-Y42, amph3) overlaps with the start of helix α1 of the ESCRT-III domain, which forms the coiled-coil hairpin with α2 (Fig. 4*B*). This third helix therefore has two hydrophobic faces: the coiled-coil interface facing α2, and the outer hydrophobic face that is involved in N-terminal hydrophobic interactions. Thus, CdvB2 has N-terminal amphipathic helices which pack in different ways to occlude their hydrophobic regions from solvent.

This analysis led us to inspect the N-terminal regions of CdvB, CdvB1, and CdvB2 of different *Sulfolobales* species to assess the conservation of their N-terminal amphipathic regions (Fig. 4*C*).

First, it was important to ensure that the predicted start codon is correct in each case. Initial sequence alignments of CdvB1 sequences indicated that the start codons of some current database entries may be misassigned (relevant here: WP_014512765.1 for *S. islandicus*, WP_011277365.1 for *S. acidocaldarius*)—as they have been predicted to have long N-terminal regions that are not present in other CdvB1s, and contain a methionine at the position that corresponds to the first residue of other CdvB1s (*SI Appendix*, Fig. S2*D*). The long leading N-terminal sequence of *S. islandicus* CdvB1 has three possible start codon positions (M1, M11, and M46). To assess which start codon is used in vivo, we expressed the three CdvB1 variants (aa 1–253, 11–253, 46–253) in *Escherichia coli* and visualized the proteins in lysates by Western blotting using an anti-SaciCdvB1 antibody (10). We compared *E. coli* lysates with the overexpressed proteins with *S. islandicus* and *S. acidocaldarius* cell lysates (*SI Appendix*, Fig. S2*E*). CdvB1^46–253^ corresponded well with the apparent size of the native *S. islandicus* CdvB1 band, indicating that the start codon is indeed misannotated in at least some database entries. Throughout this manuscript, we therefore trimmed the leading N-terminal sequence and renumber residues based on the reassigned starting methionine.

Having determined the N terminus, we could then compare regions. The N-terminal regions of these proteins have a high level of sequence diversity compared to the core ESCRT-III domain. Nevertheless, the residues forming the hydrophobic faces of the three amphipathic helices are conserved in CdvB2 orthologs. CdvB and CdvB1 sequences maintain some of the hydrophobic residues in amph1 and amph3 but lack the corresponding amph2 sequence. Helical wheel diagrams of amph1 and amph3 in *S. acidocaldarius* CdvB and CdvB1 indicate that these regions could form short amphipathic helices—similar to those observed in CdvB2 (*SI Appendix*, Fig. S3*G*).

### Interactions between CdvB Paralogues and Membranes.

CdvB, CdvB1, and CdvB2 are believed to polymerize on archaeal membranes and remodel them during cell division, driving cytokinesis. To understand this process better, we assayed how the three CdvB paralogues associate with different membranes in vitro.

**Fig. 5.**
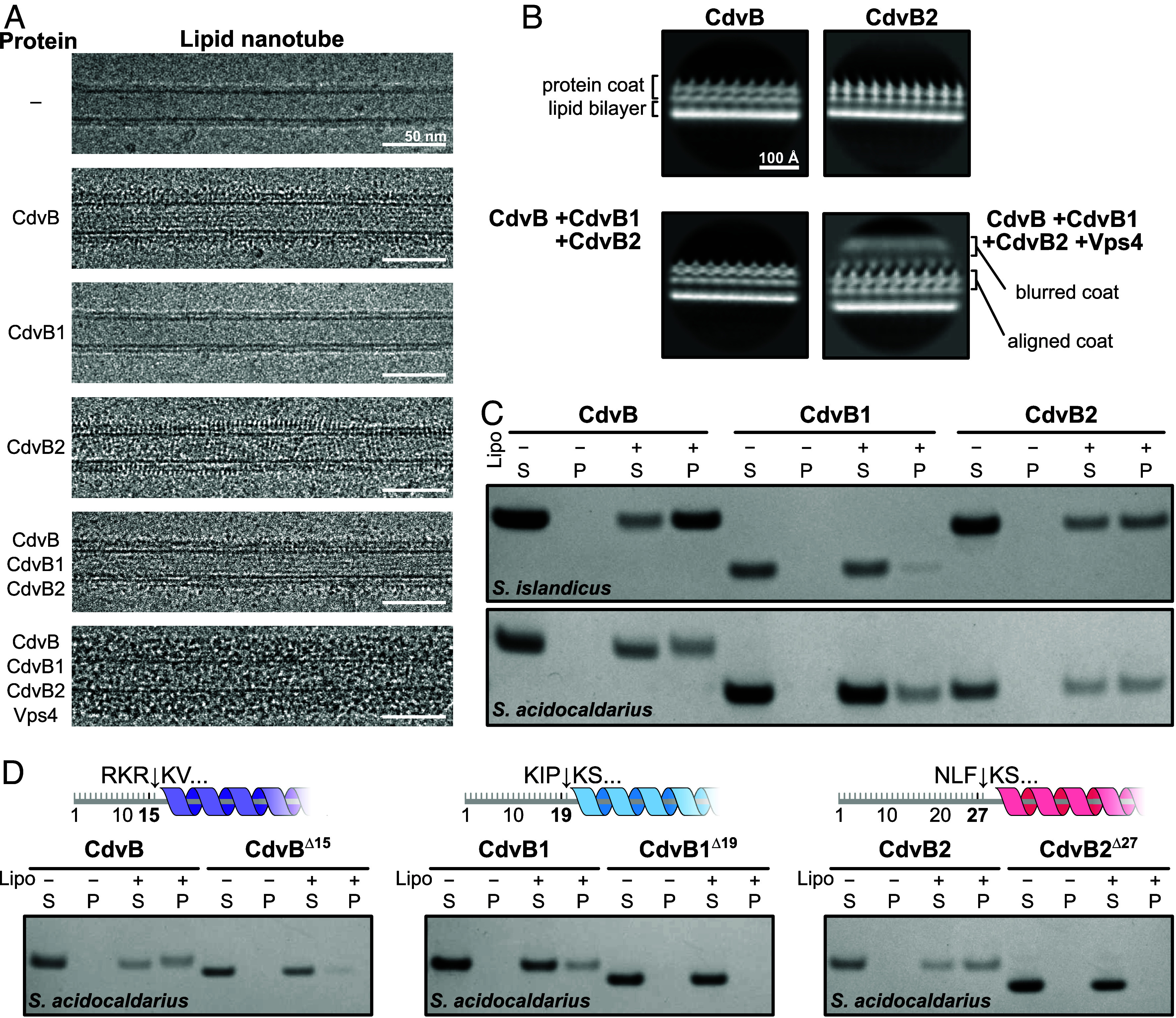
Interactions between CdvB-family proteins and lipid membranes. (*A*) Cryo-EM micrographs showing lipid nanotubes (LNTs) with different *S. islandicus* Cdv protein combinations. (*B*) 2D class averages of LNTs from panel *A*. (*C*) SDS-PAGE gels showing pelleting of CdvB, CdvB1, and CdvB2 with archaeal liposomes. (S: supernatant. P: pellet). (*D*) SDS-PAGE gels comparing pelleting with archaeal liposomes for full-length and N-terminally truncated *S. acidocaldarius* CdvB, CdvB1, and CdvB2. Truncation sites are indicated with arrows in cartoon schematics.

We incubated *S. islandicus* CdvB, CdvB1 (with the reassigned start codon) and CdvB2 in different combinations together with lipid nanotubes (LNTs) composed of *E. coli* total lipid extract and galactosyl(β)ceramide. Looking at the samples with cryo-EM (Fig. 5*A*), both CdvB and CdvB2 coated the surface of the membrane tube in a relatively regular manner. Under the same conditions, we could not see any CdvB1 bound to LNTs. Mixing all three paralogues and incubating them with LNTs produces a single-layer protein coat, although we could not determine whether it is made of a single CdvB paralogue or a mixture of all three. When the dedicated ATPase Vps4 was added to the mix and supplemented with ATP, the assembled protein coat was much thicker and less regular.

To better resolve the structures of these protein coats, we performed 2D classification and averaging of the coated nanotube edges (Fig. 5*B*). CdvB, CdvB2, and the CdvB+CdvB1+CdvB2 condition all form single-layered protein coats with ~38 Å repeats along the LNT’s long axis. The same spacing has been reported above in the monolayer-bound *S. islandicus* CdvB and soluble *S. acidocaldarius* CdvB2 filaments (Fig. 3*E* and *SI Appendix*, Fig. S2 *A* and *C*). The multilayered coat resulting from addition of Vps4 is irregular as mentioned, the 2D average showing a blurred protein coat on top of the aligned 38 Å-repeat protein layer. It remains unclear whether the additional density represents Vps4 binding to exposed MIM2 motifs, or whether it is a multilayered ESCRT-III coat that requires Vps4-mediated remodeling for assembly.

Unlike most lipids of bacteria and eukaryotes, which contain fatty acids ester-linked to a glycerol backbone, archaeal lipids contain ether-linked isoprenoid chains. Membranes of thermophiles such as *Sulfolobus* additionally have a high proportion of membrane spanning tetraether lipids, which likely confer greater thermal stability on the membrane (27). Since LNTs were made with bacterial lipids, we wanted to evaluate whether archaeal lipids alter CdvB paralogue membrane recruitment.

We generated archaeal liposomes using lipids extracted from *S. acidocaldarius* and used them to perform liposome pelleting assays. After an initial spin that removed protein aggregates and any preformed filaments, we incubated the soluble proteins with archaeal liposomes for 15 min and pelleted the liposomes, including any associated protein. We performed the experiment using both *S. islandicus* and S*. acidocaldarius* proteins.

The results of these pelleting assays were effectively consistent with LNT binding. CdvB and CdvB2 efficiently pelleted with archaeal liposomes, while CdvB1 remained mainly in the soluble fraction (Fig. 5*C*). This was consistent across the two species. The fact that these proteins exhibit the same binding profile in presence of *E. coli* and *S. acidocaldarius* lipids indicates that the CdvB paralogues are not selective for lipid backbones or tails when polymerizing on membrane.

### N-Terminal Amphipathic Helices Are Required for Efficient Membrane Recruitment.

N-terminal amphipathic helices have shown to be required for membrane binding and remodeling in eukaryotic ESCRT-IIIs (28, 29). To probe how CdvB proteins interact with the membrane, we tested the role of the N-terminal amphipathic helices identified above (Fig. 4 and *SI Appendix*, Fig. S3*G*). Having established an assay for CdvB membrane recruitment, we assessed the role of the N-terminal amphipathic helices in this process.

As we initially found short amphipathic helices in the *S. acidocaldarius* CdvB2 structure, we performed this experiment with *S. acidocaldarius* proteins as a model. We truncated the first 15 residues of CdvB (CdvB^∆15^), 19 in CdvB1 (CdvB1^∆19^), and 27 residues of CdvB2 (CdvB2^∆27^), removing amph1 and amph2 (amph3 of CdvB2 was left intact because it corresponds with the start of the core ESCRT-III domain). The truncated proteins were then tested in the membrane binding assay. In each case, the N-terminal truncation exhibited a drastic reduction in liposome binding (Fig. 5*D*). While CdvB1^∆19^ still showed a faint band in the pellet fraction, membrane binding was completely abolished in CdvB^∆15^ and CdvB2^∆27^. This indicates that the N-terminal regions are essential for CdvB paralogues to associate with membranes.

### CdvB2 on Lipid Nanotubes.

**Fig. 6.**
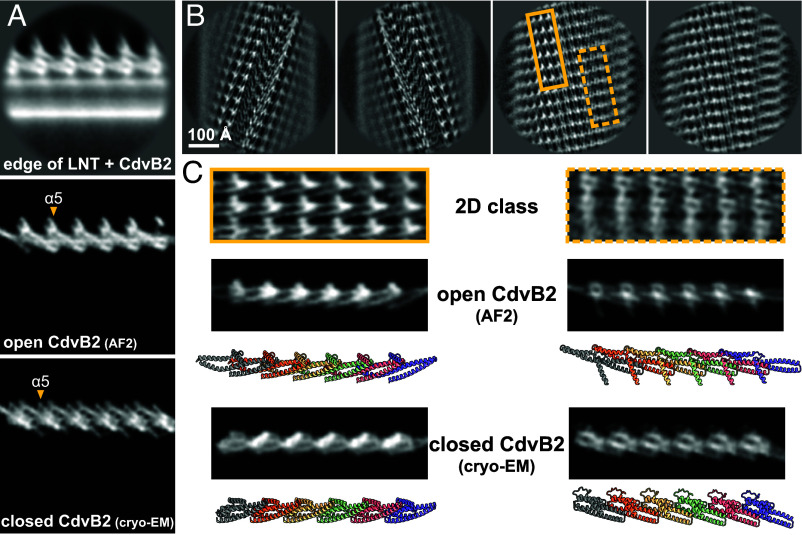
Membrane-bound CdvB2. (*A*) 2D class average of LNT edges coated with *S. acidocaldarius* CdvB2 (*Top*) alongside simulated 2D projections of open (AlphaFold2) and closed (cryo-EM, this work) filaments. To simulate projections, 10 Å resolution density maps were generated from atomic models with ChimeraX *molmap* and projected using relion_project. (*B*) 2D class averages of LNTs coated with CdvB2 after membrane signal subtraction. Two main types of high-resolution classes contained seams (*Left*) or continuous protein coats (*Right*). (*C*) Two regions of the continuous coat from (*B*) compared with simulated 2D projections of open and closed CdvB2 filaments. Corresponding molecular models are shown for each simulated projection.

To assess whether full-length CdvB2 polymers undergo a conformational change upon membrane binding, we prepared LNTs incubated with *S. acidocaldarius* CdvB2 and imaged them by cryo-EM (*SI Appendix*, Fig. S4*A*). To inspect the fine structure of the coat, we performed 2D classification on protein-coated LNT edges. The classes showed spiked edges (Fig. 6*A* and *SI Appendix*, Fig. S4*B*), similar to the *S. islandicus* samples. To assess whether *S. acidocaldarius* CdvB2 adopts a closed or open conformation in this context, we generated simulated 2D projections of closed and open CdvB2 filaments based on our cryo-EM structure and AlphaFold2 (30, 31), respectively. When directly compared, experimental 2D classes of the protein edge more closely match the simulations in the open conformation (Fig. 6*A*).

To better assess the CdvB2 conformation in the context of a membrane bound polymer, we processed a second dataset of CdvB2 on LNTs. To improve alignment we subtracted the dominating membrane signal from micrographs, as implemented in ref. 32, borrowing a technique from the field of microtubule-associated proteins (33). This greatly improved protein alignment (*SI Appendix*, Fig. S4*G*), enabling us to resolve 2D classes to secondary structure resolution (Fig. 6*B*). Two main types emerged: some had a continuous protein coat while others had a prominent seam. To further assess the conformation of the membrane-bound CdvB2, we compared different regions of the tubular protein array with simulated 2D projections of closed- and open-form filaments from equivalent angles (Fig. 6*C*). The experimental averages appeared more similar to the open CdvB2 model than the closed-form filament. These data reinforced the conclusion that membrane binding triggers CdvB2 to polymerize in the open conformation.

We noted that in some of these images we observed nonmembrane bound CdvB2 filaments in the background. All were low-twist filaments with monomers in the closed conformation, indistinguishable from the low-twist CdvB2 structure solved earlier (*SI Appendix*, Fig. S4 *C*–*F*). This implies that polymerization on membrane induces the protein to undergo a conformational switch from the closed to the open form.

## Discussion

In this work, we provide a structural and biochemical framework for understanding Cdv-based cell division. We reveal how elements of the core machinery assemble into polymers and how they engage with membranes to drive division.

CdvA, the non-ESCRT component, has a PRC domain and a three-helix coiled-coil. It forms antiparallel double-stranded helical filaments that are built from dimeric subunits and stabilized by hydrophobic interactions between the coiled-coil domains. This double filament architecture is consistent with earlier observations of *M. sedula* CdvA by negative stain electron microscopy (15, 34). While it was reported that *M. sedula* CdvA requires DNA for polymerization, we observed none in our cryo-EM sample or the final structure. PRC domains mediate lateral cross-strand interactions, providing a structural explanation for past observations that CdvA lacking this domain fails to polymerize (35). CdvA is thought to associate with the membrane in cells (8, 12), but filaments show no binding in vitro (34) and we observed no putative membrane binding regions on the filament surface. Thus, we speculate that CdvA requires additional, yet-to-be-identified partners that activate it or tether it to membranes in vivo.

The helical nature of the CdvA filament seems incompatible with its ring-forming function in cell division, which is thought to require membrane association. Possible resolutions include: the filament reassembles to form an alternate structure when membrane-bound (as observed here for CdvB2); membrane attachment is not continuous but instead periodic, occurring where the helical twist allows it; or that CdvA does not make a complete ring but instead forms short discontinuous filaments, similar to FtsA and MreB in bacterial cell division and elongation, respectively (36). Further studies will be needed to clarify CdvA’s enigmatic role in cytokinetic ring initiation.

Our analysis of the Sulfolobales ESCRT-III proteins showed that CdvB and CdvB2 adopt the canonical ESCRT-III fold, experimentally confirming their evolutionary and functional placement within the ESCRT-III protein superfamily (37). Notably, our monomeric CdvB structures reveal both a closed and a previously undescribed semiopen conformation. This suggests a possible conformational intermediate along the transition to fully open, polymer-forming state seen in other ESCRT-IIIs.

CdvB formed flat spirals on lipid monolayers, reminiscent of those formed by yeast Snf7 and *Caenorhabditis elegans* Vps32 (38, 39). All three are recruited in the initial stages of ESCRT-III-based membrane remodeling, indicating that early components may form flat spirals which then deform upon recruitment of later components, in line with proposed models (40).

We show that soluble, lipid-free CdvB2 filaments consist entirely of subunits in the closed state, a unique example of a full-length ESCRT-III filament adopting this conformation in a homopolymer. Truncated IST1, a eukaryotic ESCRT-III protein, was previously shown in closed conformation when copolymerized with CHMP1B or when mutated (20, 41), leading to the suggestion that some ESCRT-IIIs may function solely in this form. For CdvB2, however, membrane binding appears to trigger a conformational switch to the open state. Thus, while CdvB2 remains closed in solution, it adopts an open state when polymerized on membranes. This challenges a model in which polymerization alone triggers the closed-to-open switch.

Our analysis also revealed that the short N-terminal amphipathic helices in CdvB paralogues mediate membrane binding and are essential for recruitment in vitro. Amphipathic helices are common features of many membrane-associated proteins (42). They have also been observed in both in eukaryotic (19, 28, 29) and bacterial ESCRTs (43–46), where they bind membrane and are essential for proper function. In *Sulfolobus*, these amphipathic regions are notably short (9–11 residues) and, in the case of CdvB2, there are two of them. Despite their size, they are clearly sufficient to mediate membrane binding, likely aided by avidity effects in the polymer form.

It has been reported that the three CdvB paralogues are recruited sequentially in vivo and localize to distinct regions of the constricting division neck (10). Although their ESCRT-III domains are highly conserved, the N-terminal membrane-targeting regions show much greater sequence diversity. These differences may underlie their differential recruitment, distinct functions, membrane affinities, or localization on the dividing cell membrane (10).

In addition to our Cdv structures, other archaeal ESCRT-III assemblies have been reported in Asgard archaea. They form straight filaments (32) or hollow tubes (47), both with open-form subunits. This variety of morphologies in archaeal ESCRT assemblies further highlights the family’s structural plasticity. Asgard ESCRTs were also shown to interact with negatively charged phospholipids. This is consistent with our observations that head group charge, rather than lipid tail chemistry, plays an important role in recruitment of archaeal ESCRT-IIIs.

Future research should focus on the mechanism of membrane remodeling, using both in vitro and in vivo approaches. Reconstituting the constriction machinery inside liposomes and imaging division rings with fluorescence microscopy and cryo-EM would reconcile dynamic and structural information about the mechanism of ESCRT-III-dependent cell division. In addition, it will be important to image cell bridges by cryoelectron tomography (cryo-ET) (10) at different stages of division to assess the structure of these filaments in their native context. For this, we need mutants that trap the dividing cells at different constriction states. Currently one such mutant exists, Vps4^E209Q^, which causes accumulation of dumbbell-shaped cells in the final stage of division (10). Now, structures of other Cdv proteins allow rational design of other mutants, such as nonpolymerizing CdvA or mutations that lock the different ESCRT-IIIs in closed or open states. Observing how these mutants perturb division by live cell imaging, immunofluorescence, or cryo-ET would help to build a more complete model of how these proteins form large ring structures which constrict into smaller and smaller radii, leading to abscission.

One possible model, based on previously reported dome-like ESCRT-III assemblies (37, 46), predicts that each additional ring (or rung of spiral) assembles with decreasing radius relative to the last. A second model based on membrane-coated helical tubes (19) and computational modeling (10) would predict constriction by (Vps4-mediated) remodeling and removal of subunits from the constricting filament. Because division rings constrict over orders of magnitude from ~1,000 nm diameter to around ~10 nm, several different ESCRT-III proteins may be required (for example B, B1 and B2) to accommodate the greatly different curvatures.

Overall, our data support a model in which ESCRT-III proteins in archaea operate though the same fundamental principles as their eukaryotic and bacterial counterparts, downstream of archaeal specific CdvA. These ESCRT-III proteins assemble into polymers, engage via their N-terminal amphipathic helices with the membrane, which induces them to undergo conformational transitions important for their function in membrane remodeling.

## Materials and Methods

### Bacterial Plasmids.

A list of protein expression plasmids is given in *SI Appendix*, Table S4.

### Expression Plasmid Construction.

All expression plasmids were constructed in a pOPINS backbone with N-terminal 6His-SUMO. For SiCdvA, SiCdvA^ΔC^, and SaciCdvA constructs, the 6His-SUMO tags were omitted.

Synthetic genes for *S. islandicus* CdvA (AGJ62618.1), CdvB (WP_012711316.1), CdvB1 (WP_014512765.1), CdvB2 (WP_009992298.1), and Vps4 (WP_ 012711317.1) were ordered from Eurofins. gBlocks for *S. acidocaldarius* CdvA (WP_011278210.1), CdvB (WP_011278209.1), CdvB1 (WP_011277365.1), and CdvB2 (WP_011278248.1) were ordered from Integrated DNA Technologies. All were cloned into a pOPINS backbone using NEBuilder HiFi DNA assembly mix (NEB). Plasmids were transformed into *E. coli* DH5α (Thermo Fisher Scientific) and verified by Sanger sequencing (Eurofins). Point mutations and deletions were introduced by side-directed mutagenesis using NEB’s Q5 Site-Directed Mutagenesis Kit.

### SiCdvB1 Start Codon Assignment.

Three possible start codons were identified in the SiCdvB1 sequence (NCBI accession WP_014512765.1). Three untagged SiCdvB1 variants (stating positions M1, M11, and M46) were cloned into *E. coli* C43(DE3), grown in 100 mL of 2× TY medium with 50 µg/mL kanamycin, induced with 1 mM isopropyl-β-D-thiogalactoside (IPTG), and expressed for 4 h at 37° C. Cells were harvested by centrifugation.

#### Archaeal growth.

*S. islandicus* was cultured in DT medium (48), and S. *acidocaldarius* in Brock medium (49). Both were grown aerobically with stirring, at 75° C and pH 3.0 to 3.5 until late exponential phase.

#### Western blotting.

Crude lysates separated on NuPAGE 4 to 12% Bis-Tris gels (Invitrogen). Samples were transferred to a nitrocellulose membrane and blocked with 5% milk and PBS with 0.2% Tween20 (PBST) for 1 h, and probed overnight at 4° C with α-SaciCdvB1 primary antibody (10) (1:10,000) in 5% milk and PBST. After three PBST washes, membranes were incubated in 5% milk and PBST with α-chicken secondary (1:10,000) for 1 h, and a final 15 min PBST wash. The blot was imaged in a Gel Doc XR+ (Bio-Rad, 1.5 s exposure).

### Protein Expression and Purification.

All plasmids were transformed into *E. coli* C43 (DE3) cells for protein expression and grown in 2× TY medium supplemented with kanamycin (50 µg/mL for *S. islandicus*, 30 µg/mL for *S. acidocaldarius*). After protein expression, cells were collected by centrifugation for 20 min at 4,000×*g* at 4° C, flash frozen in liquid nitrogen, and stored at −80° C.

Selenomethionine-substituted proteins were also expressed in *E. coli* C43(DE3) as in ref. 50, using a protocol modified from ref. 51. Briefly, feedback inhibition of methionine synthesis was achieved by growing cells in MOPS minimal media supplemented with amino acids prior to induction with IPTG. For 12 L of culture, the amino acid mix contained 1.2 g of lysine, threonine, phenylalanine, 0.6 g of leucine, isoleucine, valine, and 0.6 g of L-selenomethionine, mixed and portioned to 0.5 g per liter of medium.

Purification steps were done at room temperature (RT) unless specified. Cells were lysed either using a cell disruptor at 25 kPsi (Constant Systems) or by sonication (15 s on, 10 s off, 70% output). Detailed purification protocols for all purified constructs are found in *SI Appendix*, *Extended Materials and Methods*. Purified proteins were aliquoted, flash-frozen in liquid nitrogen, and stored at −80° C.

### Crystal Structure Determination.

Crystallization conditions were found using our in-house crystallization facility (52). Many hundreds of commercially available screening conditions were tested for each sample, using vapor-diffusion setups in sitting drop MRC crystallization plates, mixing 100 nL of sample and 100 nL of reservoir. All crystallization setups were incubated at 20° C. Full details of crystallization conditions and structure determination protocols can be found in *SI Appendix*, *Extended Materials and Methods*.

### Filament Sample Preparation and Cryo-EM Data Collection.

#### SiCdvA^Δ^*^C^*.

3 µL of SiCdvA^ΔC^ at 0.05 to 0.1 mg/mL in buffer A1 was applied to glow-discharged 400-mesh R 2/2 copper grids (Quantifoil), immediately blotted and plunged into liquid ethane using a Vitrobot mark VI (Thermo Fisher Scientific) at RT and 100% humidity. Data were collected on a Titan Krios transmission electron microscope (Thermo Fisher Scientific) operating at 300 keV and equipped with a K2 electron detector (Gatan) in counting mode. EPU software (Thermo Fisher Scientific) was used to automate collection of 2,594 micrographs, with a dose of 1 e^−^/Å/s fractionated over 40 frames, nominal pixel size 1.068 Å/px, defocus range of −0.7 to −3.5 μm.

#### SaciCdvB2.

3 µL of SaciCdvB2 at 0.4 mg/mL in buffer BB6 [50 mM MES(NaOH); 50 mM NaCl; pH 6.0] was applied to glow-discharged 200-mesh gold R2/2 Quantifoil grids (Quantifoil), blotted, and plunged into liquid ethane using a Vitrobot mark VI at 100% humidity and RT. Data were collected on a Titan Krios at 300 keV with a Falcon 4 electron detector (Thermo Fisher Scientific) in electron counting mode. EPU software was used to automate acquisition of 3,589 movies, 5 s exposures, total dose 35.86 e^−^/Å^2^ across 40 movie frames at a nominal pixel size of 0.824 Å/px. Defocus ranged between −1.0 to −2.6 µm.

### Helical Processing.

The SiCdvA^ΔC^ and SaciCdvB2 filament structures were solved using helical reconstruction (53) in RELION-3 (54) and RELION-4 (55), respectively. See *SI Appendix*, *Extended Materials and Methods* for full processing details.

### Cryo-EM Model Building.

All cryo-EM volumes and atomic model figures were prepared with ChimeraX (v1.7.1) (56)

#### SiCdvA^Δ^*^C^*.

A dimer of SiCdvA^ΔC^ from the crystal structure was rigidly docked into the cryo-EM map, and refined in Coot and PHENIX real-space refine.

#### SaciCdvB2.

For SaciCdvB2 models, an ab initio model was built in the low-twist filament map using ModelAngelo (57). Missing regions were modeled with *Coot* (v.0.9.8.2) (58) and the ISOLDE plugin (59) in ChimeraX (v1.6.1), and real-space refined with PHENIX (60). Several copies of a single chain of this refined model were rigidly docked into the high-twist class A and class B maps. They were remodeled and refined as above.

### Cryo-EM of Lipid Nanotubes.

#### Lipid nanotube preparation.

LNTs were prepared with a modified protocol from ref. 61. Briefly, *E. coli* total lipid extract (Avanti Polar Lipids) and D-galactosyl-β-1,1′ N-nervonoyl-D-erythro-sphingosine (Galactosyl(β) ceramide, Avanti Polar Lipids) were mixed together at a 50:50 mass ratio, dried under a gentle stream of nitrogen gas, and left in a desiccator overnight to remove residual solvent. The lipid film was rehydrated in 50 µL of buffer BB6 to a final lipid concentration of 2 mg/mL and incubated at RT for 30 min, then placed on a rotary mixer at RT for 15 min, and finally transferred to a sonication bath for 4 min. Once formed, the LNTs were stored at 4° C.

#### LNT cryo-EM sample preparation and data collection.

LNT solution was mixed with various combinations of *Sulfolobus* proteins and incubated for 1 h at 37° C. For most samples, final concentration of LNTs and of each protein component was 0.2 mg/mL, at pH 7. For samples containing Vps4, 2 mM ATP and 4 mM MgCl_2_ were added. For LNT-only grids, their concentration was 1 mg/mL. Grids of each sample were prepared as above, freezing 3 µL of solution on an R2/2 200-mesh gold Quantifoil or 400-mesh UltrAuFoil grid in a Vitrobot mark IV. They were imaged in a Glacios at 200 keV with a Falcon III detector.

For the LNT-SaciCdvB2 sample, dataset I was collected on a Titan Krios with a K3, recording 10,599 movies at superresolution and 2× binning on the fly. A dose of 35.28 e^−^/Å^2^ was delivered over 1.3 s exposures and fractionated over 70 frames, across a defocus range of −1.0 to −2.6 µm. For dataset II, 23,891 movies were collected on a Titan Krios with a K2 detector and BioQuantum energy filter (Gatan). A total dose of 40 e^−^/Å^2^ was fractionated over 43 frames, and a defocus range of −1.2 to −2.7 µm was applied.

#### LNT cryo-EM data processing.

For dataset I, particles were extracted at 38 Å spacing and underwent several rounds of 2D classification in RELION. Soluble filaments were picked with a crYOLO filament model and processed similarly to purified CdvB2 filaments described earlier. For dataset II, we subtracted membrane signal at the micrograph level as implemented in ref. 32, using scripts for tubulin lattice subtraction (33). The resulting subtracted particles were 2D classified in CryoSPARC and RELION. See *SI Appendix*, *Extended Materials and Methods* for full details.

### Pelleting Assay.

#### *S. acidocaldarius* lipid extraction.

*S. acidocaldarius* was grown as described earlier, harvested, and freeze-dried. Archaeal lipid extraction protocol was modified from ref. 62. 4.5 g of cells were extracted in 400 mL of a chloroform–methanol (CHCl_3_–MeOH, 1:1 v/v) mixture in a Soxhlet extractor overnight. The lipid extract was dried and resuspended in 20 mL of H_2_O-MeOH mixture (1:1 v/v) and extensively sonicated for a few hours in a bath sonicator to resolubilize the extracted lipids. The resulting solution was loaded on a Sep-Pak C18 20 cc Vac cartridge (Waters) used under reduced pressure. The column was washed with 250 mL H_2_O-MeOH (1:1 v/v) and lipids eluted with 250 mL of CHCl_3_-MeOH-H_2_O (1:2.5:1 v/v/v). The solution was dried in a rotary evaporator. The lipids were washed by resuspending in CHCl_3_-MeOH-H_2_O (65:25:4), aliquoting into smaller tubes, and drying in a rotary evaporator. The dry lipids were then weighed and resuspended in CHCl_3_-MeOH-H_2_O.

#### Archaeal liposome preparation.

1 mg of *S. acidocaldarius* lipid extract in CHCl_3_-MeOH-H_2_O was dried under a stream of nitrogen gas and left under vacuum overnight to remove residual solvent. The lipid film was then rehydrated with buffer BB6 to 4 mg/mL and shaken vigorously at 80 °C for 3 h, vortexing every 10 to 15 min. After 8 to 10 freeze–thaw cycles the liposomes were extruded through a 0.8 µm Whatman membrane filter using a miniextruder (Avanti) with glass gas-tight syringes (1 mL, Avanti).

#### Pelleting.

Proteins were rapidly thawed and resuspended to 0.6 mg/mL in buffer BB6. 20 µL of each sample was centrifuged in a TLA-100 rotor (Beckman Coulter) for 30 min at 100,000×*g* and 4° C to pellet any preformed aggregates and polymerized protein. 5 µL of the prespun supernatant was mixed with 30 µL of *S. acidocaldarius* liposomes and incubated at RT for 15 min before spinning for 30 min at 100,000×*g* and 4° C. The pellets were briefly washed and analyzed alongside supernatant samples on NuPAGE 4 to 12% Bis-Tris SDS-PAGE gels (Invitrogen).

### SiCdvB on Lipid Monolayer.

#### Monolayer preparation.

Lipid monolayers were prepared on electron microscopy grids using *E. coli* polar lipid extract (Avanti Polar Lipids) (63). A custom Teflon block was filled with 60 μL of buffer [50 mM HEPES(KOH); 100 mM potassium acetate; 5 mM magnesium acetate; pH 7.7]. 20 μg of lipids were dissolved in chloroform, applied on top of the buffer, and incubated for 2 min. Electron microscopy grids (CF300-Cu-UL for negative stain, 300-mesh gold R0.6/1 Quantifoil for cryo-EM) that were baked at 60° C overnight were placed on top of the lipid film, carbon side down, and incubated for 30 to 60 min. The grids were then carefully lifted, blotted from the side, then washed once with 4 μL of fresh buffer and blotted again.

#### Negative stain sample preparation and imaging.

4 μL of SiCdvB at 0.05 mg/mL (diluted with buffer BB6) was applied to monolayer grids, incubated for 30 s, and stained with 2% (w/v) uranyl formate. Grids were imaged on a Tecnai Spirit transmission electron microscope (120 keV, Thermo Fisher Scientific) with a Gatan Orius SC200W camera.

#### Cryo-EM sample preparation, data collection, and processing.

3 μL of SiCdvB at 0.15 mg/mL (diluted with buffer BB6) was applied to monolayer grids, blotted, and plunged into liquid ethane using a Vitrobot mark VI at RT and 100% humidity. Micrographs were collected on a Glacios transmission electron microscope (Thermo Fisher Scientific) operated at 200 keV and equipped with a Falcon III detector in linear mode. Movies were collected using EPU, using a total dose of 19.7 e^−^/Å^2^ fractionated across 39 movie frames at a nominal pixel size 2.55 Å/px. Defocus ranged between −3 to −5 µm in 0.4 µm increments.

Processing was done in RELION-4.0. Micrographs were motion-corrected with RELION’s MotionCor2 implementation and CTF estimated with CTFFIND4. Particles were picked with a modified version of Topaz (64, 65). Coordinates were extracted in boxes of 128 pixels and downscaled to 64 pixels for three rounds of 2D classification.

## Supplementary Material

Appendix 01 (PDF)

## Data Availability

Protein structure coordinates have been deposited in the Protein Data Bank (9S9G (66), 9S9I (67), 9S9J (68), 9S9K (69), 9S9H (70), 9S97 (71), 9S98 (72), and 9S99 (73)). Cryo-EM maps have been deposited in the Electron Microscopy Data Bank (EMD-54673 (74), EMD-54674 (75), EMD-54675 (76), EMD-54678 (77)). All other data are included in the manuscript and/or *SI Appendix*.
