# Supplementary material for: Molecular structure of the ESCRT-III-based archaeal CdvAB cell division machinery
Source: Proc Natl Acad Sci U S A. 2026 Jan 16;123(3):e2525941123. doi: 10.1073/pnas.2525941123 (PMC12818579; doi:10.1073/pnas.2525941123)
Supplement: Supplementary file 1 — Appendix 01 (PDF) [file pnas.2525941123.sapp.pdf]

Supporting Information for

Molecular structure of the ESCRT-III-based archaeal CdvAB cell division machinery

Tina Drobnič[1], Ralf Salzer[1], Tim Nierhaus[1], Margaret Jiang[1], Dom Bellini[1], Astrid Steindorf[2], Sonja-Verena Albers[2], Buzz Baum[1], Jan Löwe*[1]

[1]Medical Research Council Laboratory of Molecular Biology, Cambridge, United Kingdom

[2]Molecular Biology of Archaea and CIBSS, Faculty of Biology, University of Freiburg, Freiburg, Germany

*Corresponding author:

Jan Löwe, MRC Laboratory of Molecular Biology, Francis Crick Avenue, Cambridge CB2 0QH, UK; email: jyl@mrc-lmb.cam.ac.uk

This file includes:

Extended Materials and Methods
Figures S1 to S4
Tables S1 to S4
References

Extended Materials and methods

Protein expression and purification

SiCdvA and SiCdvA$^{\Delta C}$

12 L of growth media was inoculated with overnight culture at 1:50 and grown at 15°C, shaking at 200 rpm until optical density OD_{600} ~0.1. Protein expression was induced with 1 mM IPTG and incubated at 15°C for 4-7 days, reaching OD_{600} ~6-7.

Cell pellets were dissolved in buffer A1 (30 mM Tris/HCl; 150 mM NaCl; 4 mM TCEP; 0.5% CHAPS (w/v); pH 8.0) with DNAse (Sigma-Aldrich) and 4 cOmplete EDTA-free protease inhibitor tablets (Roche). Cells were lysed by sonication and spun for 15 min at 20,000 *g* at RT to pellet debris. The supernatant and hard pellet were discarded. The wobbly pellet was washed 3-4 times by resuspending in buffer A1 and pelleting at 20,000 *g* for 15 min. CdvA filaments were spun at 15°C for 1 h at 45.000 rpm in a 45Ti rotor (Beckman Coulter) and resuspended in 50 mL of 1 M CHES (pH 9.5) to dissolve filaments. After 30 minutes at RT, the solution was spun at 4°C for 30 min at 35,500 *g* in a JLA16.250 rotor (Beckman Coulter) to remove precipitated protein. Supernatant was dialysed against buffer A1 without CHAPS in 3 kDa Slide-A-Lyzer dialysis cassettes (Thermo Scientific), stirring overnight at RT. The re-formed filaments were spun at 15°C for 3-5 h or overnight at 45,000 rpm and resuspended in the desired volume to reach required concentrations (typically 7.5 mg/mL).

SiCdvA^{noF}

4 L of growth media was inoculated with 20 mL overnight culture and grown at 15°C, shaking at 200 rpm. Expression was induced with 1 mM IPTG at OD_{600} ~0.3 and continued at 15°C for 24 h.

Cells were resuspended in 100 mL buffer A2 (30 mM CHES; 100 mM NaCl; 4 mM TCEP; 1% CHAPS (w/v); pH 9.0) and protease inhibitor. The cells were then lysed by sonication and the solution was topped up to 400 mL (final CHAPS concentration 0.25%) and spun at RT for 15 min at 16,250 g to pellet debris. Supernatant was collected and imidazole added to 5 mM, followed by incubation with 10 mL of Super Cobalt NTA affinity resin (Generon) for 45 min. The protein was loaded onto a gravity flow column and washed with buffer A2. Protein was eluted stepwise in buffer A2 with imidazole at concentrations: 25 mM, 50 mM, 75 mM, 100 mM, and 250 mM. Elution fractions containing pure CdvA were pooled, concentrated, and further purified on a HiLoad 16/600 Superdex 200 pg gel filtration column (Cytiva) in buffer A2. Peak fractions were pooled and concentrated to ~50 mg/mL.

SiCdvB, SiCdvB1^{M46}, and SiCdvB2

12 L of growth media was inoculated with 60 mL overnight culture and grown at 37°C, shaking at 150 rpm until OD_{600} 0.7-1.0. The temperature was reduced to 18°C and protein expression was induced with 1 mM IPTG, expressing for 20-24 h.

Cells were dissolved in buffer B1 (30 mM Tris/HCl; 150 mM NaCl; pH 8.8), DNAse, and protease inhibitor. Cells were lysed by sonication and centrifuged at 20°C for 20 min at 16,250 *g* to pellet debris. The supernatant was incubated with 20 mL of Super Cobalt NTA resin (Ni-NTA Agarose resin (Qiagen) for SiCdvB) stirring at RT for 25 min. The slurry was loaded on a gravity flow column and washed multiple times with buffer B1 and buffer B1 containing 5 mM imidazole. The protein was eluted 3x with 20 mL buffer B1 with 400 mM imidazole. Elution fractions were pooled in 3 kDa Slide-A-Lyzer dialysis cassettes together with homemade SENP1 (1) and dialysed overnight against buffer B2 (50 mM Tris/HCl; 100 mM NaCl; 4 mM TCEP; pH 8.0). The next day, dialysed sample was incubated in a 70°C water bath for 10 min and immediately spun for 3 min at 16,200 *g* to remove precipitates. This heat-inactivation step was skipped for SiCdvB1^{M46}. The supernatant was incubated for 1 h with equilibrated Ni-NTA resin and ran over a gravity column, capturing the flowthrough to collect SiCdvB without affinity tags. 1 M CHES (pH 9.5) was added to a final concentration of 100 mM and the sample was

concentrated and further purified on a HiLoad 16/600 Superdex 75 pg gel filtration column (Cytiva) equilibrated with buffer B3 (30 mM CHES; 150 mM NaCl; pH 9.5), and clean fractions concentrated to ~9.5 mg/mL.

SiCdvB$^{ΔC\ I69M\ I125M}$ with selenomethionine

12 L of pre-warmed MOPS media supplemented with 50 µg/mL kanamycin was inoculated with overnight culture and grown at 37°C, 200 rpm for ~4 h until OD_{600} 0.6-0.7. Then, amino acid mix was added and temperature reduced to 18°C. After 30 min, protein was induced with 1 mM IPTG and expressed overnight before harvesting. The protein was purified as SiCdvB.

SaciCdvB, SaciCdvB1, and SaciCdvB2 and their truncations

All *S. acidocaldarius* proteins were expressed by inoculating 12 L of culture 1:100 with overnight culture and growing it to OD_{600} 0.6-0.8, 37°C with shaking at 190 rpm. Protein expression was induced with 0.5 mM IPTG and grown for a further 4 h before harvesting.

Cells were resuspended in buffer B3 with DNase, RNase, and protease inhibitor tablet. They were lysed by sonication and debris pelleted by spinning at 30,000 rpm (Ti45 rotor) for 30 min at 15°C. The supernatant was clarified by filtering through a 0.22 µm filter and imidazole was added to 20 mM. This sample was loaded onto a 5 mL HisTrap HP (Cytiva) equilibrated in buffer B3 with 20 mM imidazole. The column was washed until the UV trace was flat, and then protein was eluted in steps of 50 mM, 100 mM, 200 mM, 300 mM, 500 mM, 1000 mM imidazole. Relevant fractions were pooled and 1 mM TCEP, ~1 mg homemade GST-tagged SENP1 (1), and 1 mL of pre-washed Glutathione Sepharose 4B resin (Cytiva) were added. All subsequent steps were done at 4°C. The sample was dialysed overnight against buffer B3 with 1 mM TCEP and then ran over a gravity flow column to remove GST-SENP1. To collect cleaved protein, the sample was run over a 5 mL HisTrap HP in buffer B3 with 20 mM imidazole and the flowthrough collected. SaciCdvB was then put through an anion exchange step, which was omitted for SaciCdvB1 and SaciCdvB2. For anion exchange, a 1 mL HiTrap Q HP anion exchange column was equilibrated in buffer B4 (30 mM Tris/HCl; pH 8.9). SaciCdvB was loaded, washed, and gradient eluted to 500 mM NaCl over 35 min. All samples were then subjected to HiLoad 16/600 Superdex 75 pg column in buffer B4 (30 mM CHES; 400 mM NaCl; pH 9.5), and appropriate fractions were pooled and concentrated.

Crystal structure determination

SiCdvA^{ΔC}

was best crystallised at 7.5 mg/mL with reservoirs containing 0.282 M KH_2PO_4 and 0.094 M Tris/acetate (pH 8.5). For harvesting, crystals were cryoprotected with solutions containing 50 % reservoir solution and 50 % glycerol (v/v). Data was collected on beamline I03 at Diamond Light Source (Harwell, UK). Because the data was weak, partly because of the very long c-axis of the unit cell, data from a number of related crystals, from different conditions, were also collected and subsequently merged into one large dataset with very high multiplicity (26.2, see Table S1 for data collection statistics). Structure determination started with molecular replacement using the PRC subdomain (PDB ID 1PM3). Several copies of the PRC domain could be located using PHASER (2) and the resulting map was improved by NCS averaging using DM as part of the CCP4 package (3). Finally, 14 copies of CdvA could be located and were traced with BUCANEER (4). Manual building in MAIN (5) and refinement with phenix.refine (6) resulted in an atomic model at 2.9 Å resolution (see Table S1 for model and refinement statistics).

SiCdvA^{ΔCnoF}

SiCdvA^{ΔCnoF} was crystallised with reservoir solution containing 18 % (w/v) PEG 4000, 0.3 M sodium acetate, 0.1 M Tris pH 9.0. Drops to produce diffraction-quality crystals were mixed from 200 nl sample and 200 nl reservoir solution. The sample was at 25 to 50 g/L. Since no additional cryo protection was

needed, crystals were harvested directly. Diffraction data was collected on a MarDTB imaging plate detector mounted on an FR-E home rotating anode X-ray source (Rigaku). The structure was solved by molecular replacement (PHASER) using a monomer from the SiCdvA$^{\Delta C}$ structure determined above. Manual adjustments in MAIN and refinement with phenix.refine yielded a highly reliable structure at 2.2 Å resolution (see Table S1 for model and refinement statistics).

SiCdvB$^{\Delta C\ I69M\ I125M}$

Because SiCdvB$^{\Delta C}$ yielded crystals that diffracted to 3.5 Å, only, which made reliable structure determination difficult, two methionine residues were introduced by mutation, to enable experimental SAD (single wavelength anomalous diffraction) phasing after seleno-methionine labelling: I69 and I125 were mutated to methionine. Using this protein, SiCdvBΔC(I69M, I125M), the best small crystals were obtained using the following reservoir solutions, mixing 100 nl sample at 10 g/L with 100 nl reservoir: 5 % (v/v) 2-propanol, 1M ammonium sulphate or 0.2 M ammonium sulphate, 15 % (w/v) PEG 4000, buffer pH 3.5. The largest crystals were obtained by eventually scaling crystallisation setups to 2 µL sample plus 2 µL reservoir. Crystals were harvested in solutions mixing 30 % (v/v) glycerol with 70 % (v/v) reservoir solution. X-ray diffraction data was collected on beamline I04 at Diamond Light Source (Harwell, UK). Two different crystal forms were solved: P 41 21 2, to 2.7 Å resolution and P 21 21 21, to 2.2 Å resolution. Structure determination used the very good anomalous signal from selenium and phases were obtained using CRANK2 (7). Models were built manually in MAIN and refined with phenix.refine.

Helical processing

SiCdvA$^{\Delta C}$

Movies were motion-corrected using RELION's implementation of MotionCor2 (8). CTF parameters were estimated with CTFFIND4 (9). A subset of micrographs was manually picked for filaments, and resulting 2D classes used for reference-based autopicking in RELION.

Particles were extracted at 63 Å spacing, with a box of 320 px and 1.068 Å/px. The data underwent rounds of 2D classification, and 253,803 particles were used in 3D refinement. Simulated map from the CdvA$^{\Delta C}$ filament solved by X-ray crystallography was used as an initial model. After refining symmetry parameters through 3D refinement, particles underwent CTF refinement and Bayesian polishing. The final reconstruction reached 4.07 Å (twist 48.96°, rise -63.64 Å). The map was sharpened with standard post-processing in RELION.

SaciCdvB2

Movies were pre-processed as above. crYOLO was used to train a filament model and pick SaciCdvB2 filaments (10, 11). Helical reconstruction was done in RELION-4.0 (12, 13).

Initial 2D classifications in RELION showed a signal for 19 Å repeats, so boxes were extracted along filaments at 19 Å increments. 3x binned helical segments were extracted with a box size 142 px. Rounds of 2D classification revealed high-twist and low-twist classes. A subset of high-twist and low-twist particles were imported into CryoSPARC (14) for *ab initio* model generation (setting maximum resolution to 8 Å). Helical segments in RELION were then re-extracted at 2x binning (150 px box). Helical symmetry parameters were approximated from *ab initio* models and refined in a 3D classification job. This separated the high-twist (158,229) and low-twist (136,416) particles. Both were first refined via helical reconstruction without symmetry. Particles were again re-extracted at a box size 300 px down-sampled to 200 px and underwent several rounds of helical refinement testing different symmetry parameters. After Bayesian polishing (15) and CTF refinement (16), the low-twist filament structure was refined to 3.2 Å, applying a twist of 1.68°, rise of 38.14 Å, and C2 symmetry. The polished and CTF-refined high-twist particles underwent a round of 3D classification without alignment, which resolved

two distinct classes differing in their lumenal densities. High-twist filament class A (139,884 particles) refined to 3.9 Å (twist 176.24°, rise 18.82 Å) and class B (18,345 particles) reached 4.0 Å (twist 176.92°, rise 18.92 Å). All maps were sharpened with standard post-processing in RELION and helical symmetry applied in real-space with *relion_helix_tolbox*.

Lipid Nanotube cryo-EM data processing

Movies were processed with RELION's implementation of MotionCor2 (8) and CTF parameters were estimated with CTFFIND4 (9). For dataset I, edges of LNTs were manually picked and used as a basis for template-based autopicking. As there was a clear 38 Å repeat, boxes were extracted with 38 Å spacing. The particles underwent several rounds of 2D classification in RELION 5. A crYOLO filament model (10, 11) was trained to pick soluble filaments present in this sample. 1.7 million particles were extracted and underwent 2 rounds of 2D classification, 3D refinement without symmetry and 3D classification without alignment. A subset of random 193 thousand particles was refined with applied helical and C2 symmetry, giving a 3.4 Å map of low-twist CdvB2 with comparable helical parameters.

For dataset II, a filament model in crYOLO was trained to pick whole LNTs. Particles were extracted at 38 Å spacing but 2D classification quickly revealed that membrane signal, and not protein lattice signal, was dominating the alignment. To enable alignment of protein signal, the picked coordinates were resampled to 29 Å (out of phase with the 38 Å repeat). A rolling 2D average of these out-of-phase picks enhanced membrane signal – it was then used to subtract membrane signal from the micrographs. The subtraction was implemented as in (17), using scripts for tubulin lattice signal removal (18). 6.5 million crYOLO picks of whole LNTs were extracted from membrane-subtracted micrographs, with a box size of 256 px at 2.73 Å/px. After importing into CryoSPARC particles were split into subsets for faster 2D classification. Initial classification showed presence of two main class types, those with a seam and those without. Data was further classified to separate the two populations and re-imported into Relion for re-extraction at 2.19 Å/px and further 2D classification at finer angular sampling. In the case of classes without a seam, a rare class that showed hints of secondary structure was chosen for further processing. Additional particles were systematically extracted, shifting coordinates along the filament axis at multiples of the 38 Å filament spacing. After more 2D classification and removal of duplicate particles, this increased the total number of quality particles in classes with secondary structure features.

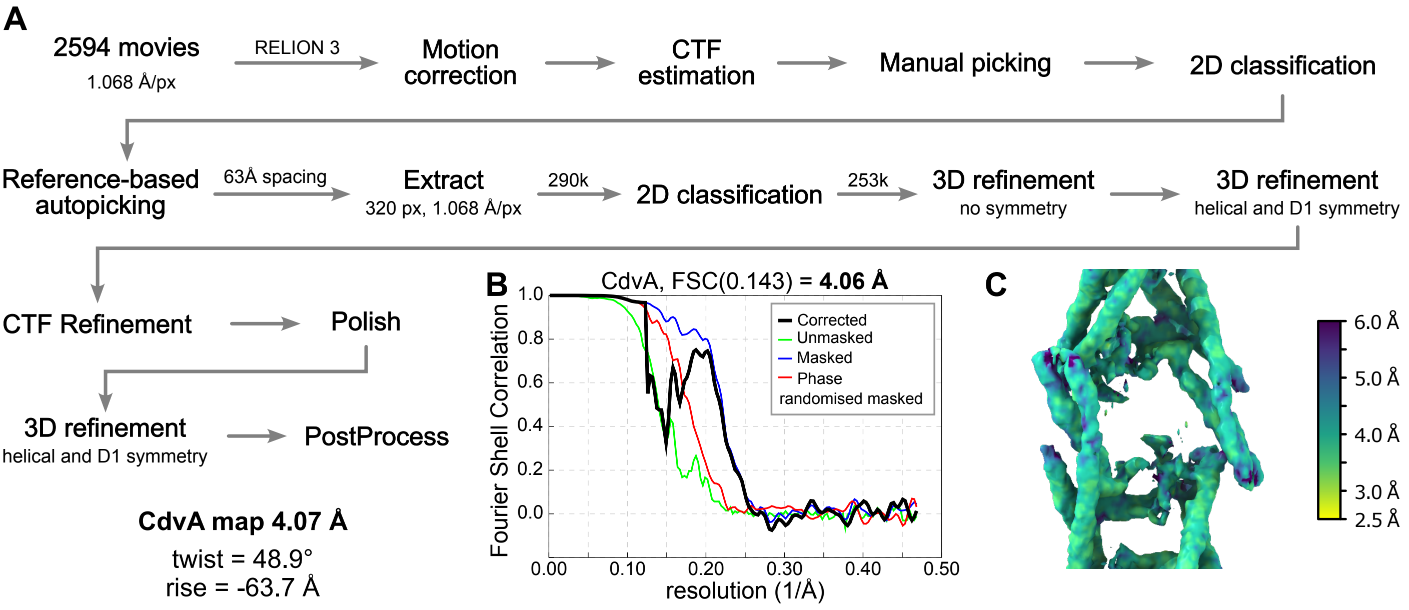

Figure S1: CryoEM of *S. islandicus* CdvA$^{\Delta C}$.

(A) Cryo-EM processing flowchart for the *S. islandicus* CdvA$^{\Delta C}$ filament structure. All steps were performed in Relion. **(B)** FSC plot and **(C)** local resolution estimations of the CdvA$^{\Delta C}$ filament.

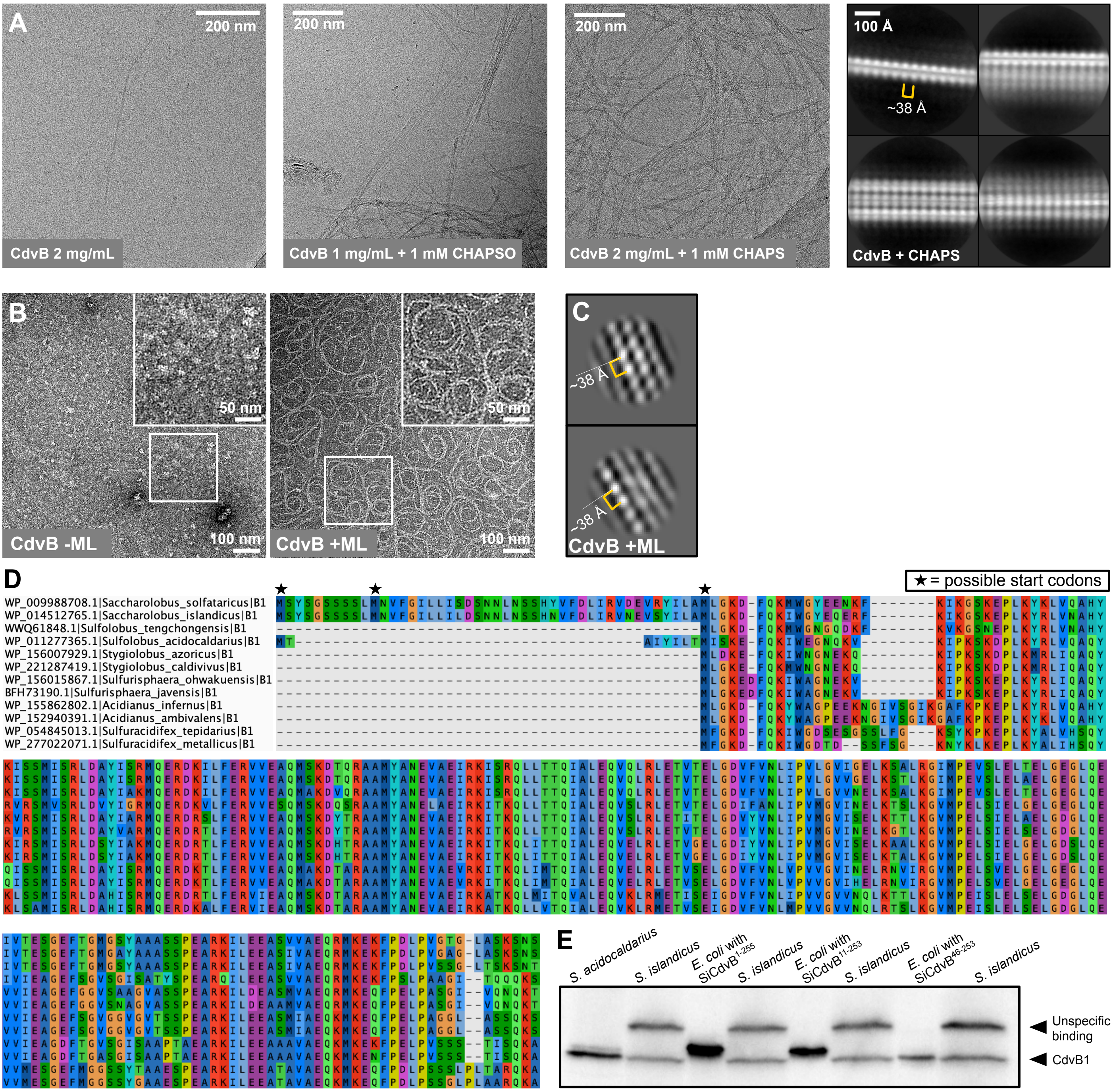

Figure S2: Polymerisation of CdvB and start codon assignment of CdvB1.

(A) Cryo-EM images of *S. islandicus* CdvB filaments and bundles under different buffer conditions. **(B)** Negative stain electron micrographs of CdvB in absence (left) and presence (right) of a lipid monolayer of *E. coli* polar lipid extract. **(C)** CryoEM 2D class averages of CdvB on lipid monolayer show ~38 Å subunit spacing **(D)** Sequence alignment of full-length *Sulfolobales* CdvB1 sequences from the NCBI protein database. **(E)** Western Blot of CdvB1 (α-SaciCdvB1 antibody) on *S. aciodocaldarius* and *S. islandicus* cell lysates, alongside *E. coli*-expressed SiCdvB1 constructs with different start codon positions. The *S. acidocaldarius* CdvB1 antibody recognises *S. islandicus* CdvB1 but also produces an unspecific signal at a higher molecular weight.

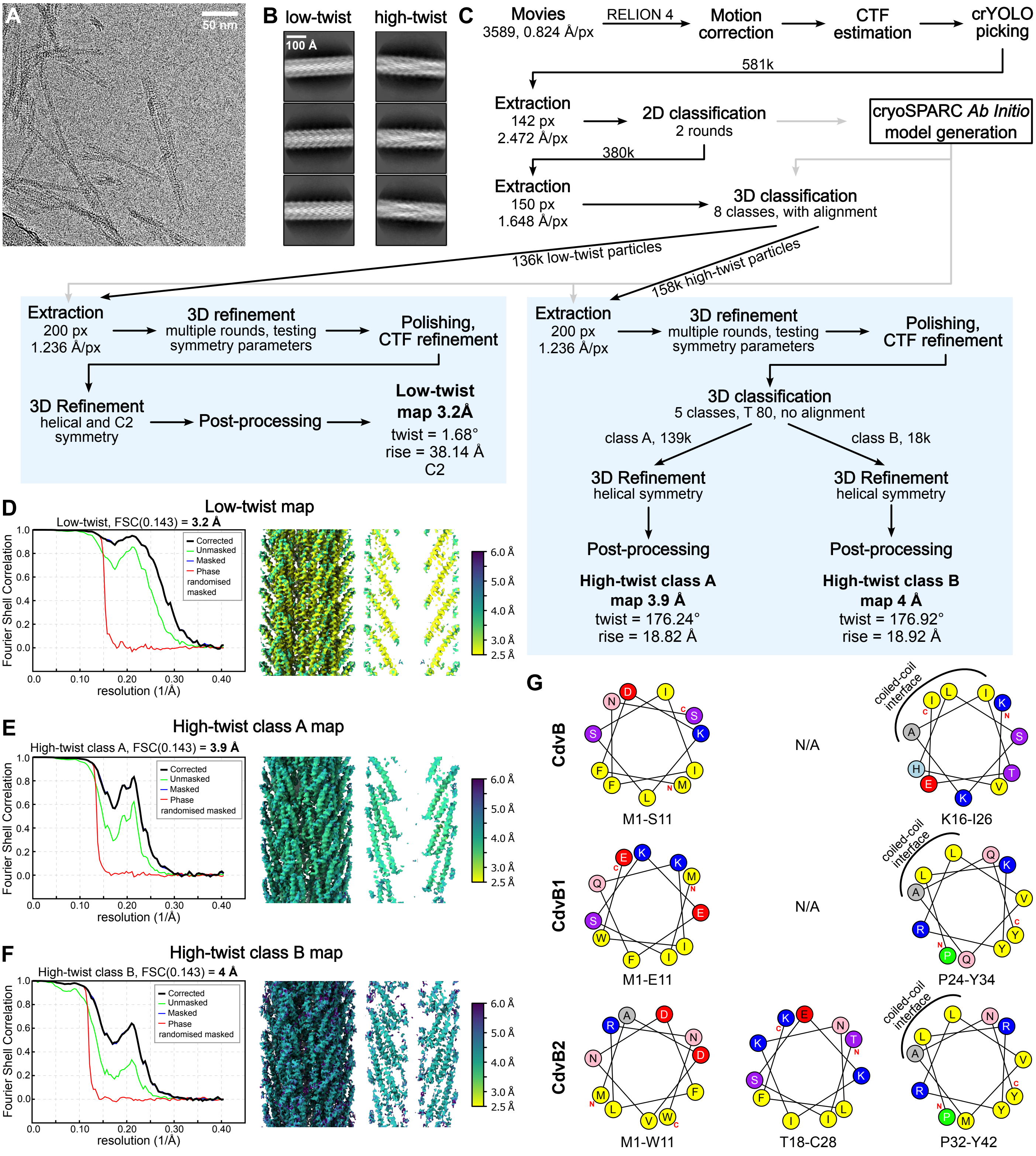

Figure S3: Cryo-EM processing of CdvB2 filaments.

(A) Representative cryo-EM micrograph of *S. acidocaldarius* CdvB2 filaments. **(B)** Example 2D class averages of high-twist and low-twist CdvB2 filaments. **(C)** Cryo-EM processing flowchart for obtaining three CdvB2 cryo-EM maps. All steps except cryoSPARC *ab initio* model generation were performed in Relion 4. **(D, E, F)** FSC plots (left) and local resolution maps (right) of the three CdvB2 maps. **(G)** Helical wheel diagrams of *S. acidocaldarius* CdvB and CdvB1 N-terminal regions that correspond to amphipathic helices of CdvB2 (shown in Figure 3H-K).

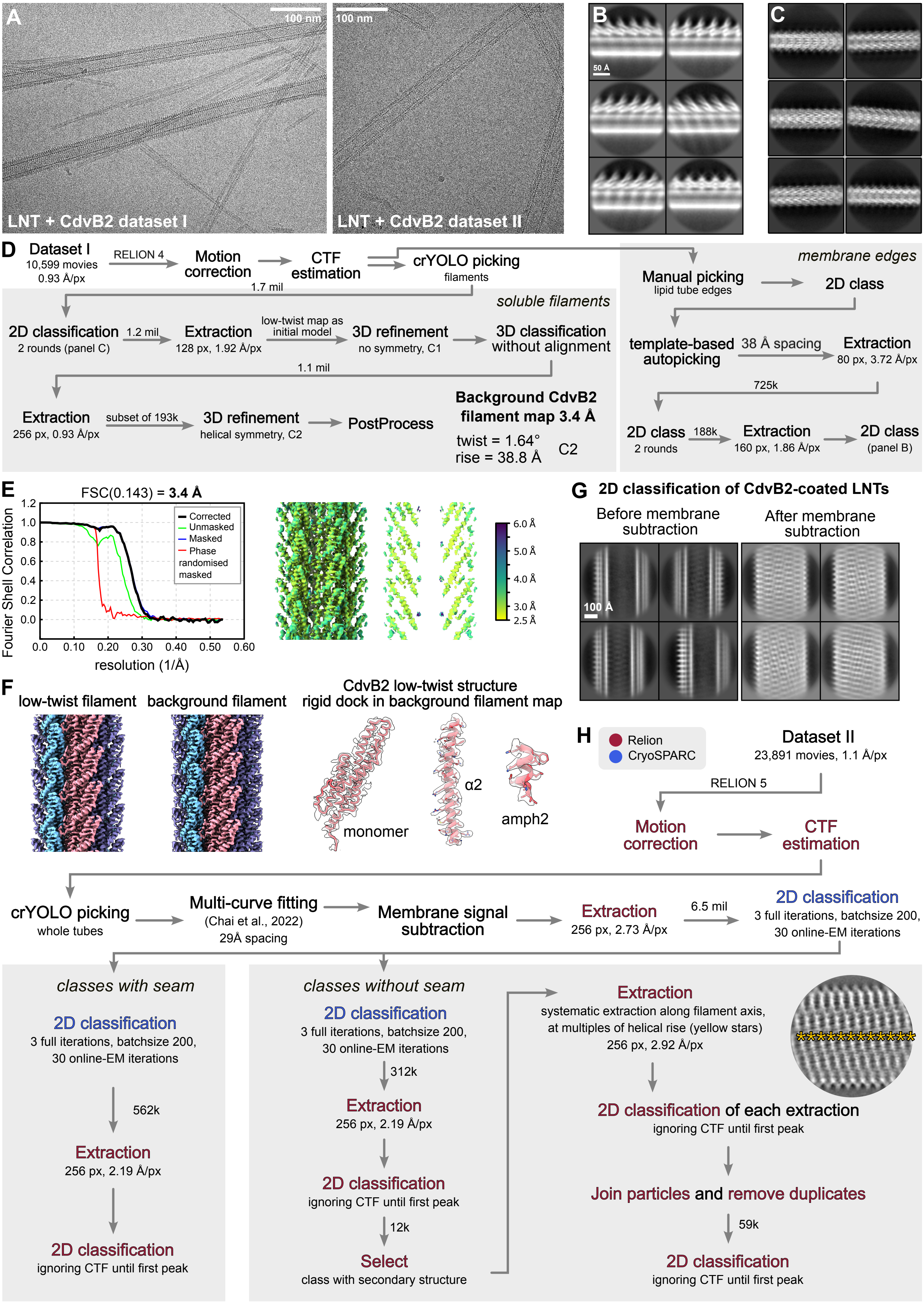
A
100 nm
LNT + CdvB2 dataset I
100 nm
LNT + CdvB2 dataset II
B
50 Å
C
D
Dataset I
10,599 movies
0.93 Å/px
RELION 4
Motion correction
CTF estimation
1.7 mil
crYOLO picking
filaments
soluble filaments
2D classification
2 rounds (panel C)
1.2 mil
Extraction
128 px, 1.92 Å/px
low-twist map as initial model
3D refinement
no symmetry, C1
3D classification without alignment
1.1 mil
Extraction
256 px, 0.93 Å/px
subset of 193k
3D refinement
helical symmetry, C2
PostProcess
Background CdvB2 filament map 3.4 Å
twist = 1.64°
rise = 38.8 Å
C2
membrane edges
Manual picking
lipid tube edges
2D class
template-based autopicking
38 Å spacing
Extraction
80 px, 3.72 Å/px
725k
2D class
2 rounds
188k
Extraction
160 px, 1.86 Å/px
2D class
(panel B)
E
FSC(0.143) = 3.4 Å
Fourier Shell Correlation
resolution (1/Å)
Corrected
Unmasked
Masked
Phase randomised masked
6.0 Å
5.0 Å
4.0 Å
3.0 Å
2.5 Å
F
low-twist filament
background filament
CdvB2 low-twist structure
rigid dock in background filament map
monomer
α2
amph2
G
2D classification of CdvB2-coated LNTs
Before membrane subtraction
After membrane subtraction
100 Å
H
Relion
CryoSPARC
Dataset II
23,891 movies, 1.1 Å/px
RELION 5
Motion correction
CTF estimation
crYOLO picking
whole tubes
Multi-curve fitting
(Chai et al., 2022)
29Å spacing
Membrane signal subtraction
Extraction
256 px, 2.73 Å/px
6.5 mil
2D classification
3 full iterations, batchsize 200, 30 online-EM iterations
classes with seam
2D classification
3 full iterations, batchsize 200, 30 online-EM iterations
562k
Extraction
256 px, 2.19 Å/px
2D classification
ignoring CTF until first peak
classes without seam
2D classification
3 full iterations, batchsize 200, 30 online-EM iterations
312k
Extraction
256 px, 2.19 Å/px
2D classification
ignoring CTF until first peak
12k
Select
class with secondary structure
Extraction
systematic extraction along filament axis, at multiples of helical rise (yellow stars)
256 px, 2.92 Å/px
2D classification of each extraction
ignoring CTF until first peak
Join particles and remove duplicates
59k
2D classification
ignoring CTF until first peak

Figure S4: Cryo-EM of lipid nanotubes (LNTs) coated with *S. acidocaldarius* CdvB2.

(A) Example micrographs of two collected datasets of LNTs and CdvB2. **(B)** 2D class averages of the edges of LNT membranes coated with CdvB2 in dataset I. **(C)** 2D class averages of CdvB2-only filaments in dataset I. **(D)** Cryo-EM processing pipeline for soluble background CdvB2 filaments and CdvB2-coated LNTs in dataset I. All steps were performed in Relion 5 unless specified. **(E)** FSC plot (left) and local resolution map (right) of the soluble background filament map. **(F)** Low-twist CdvB2 filament map from Figure 3 (left) and the soluble background filament map (middle). The modelled low-twist structure rigidly docked into the background filament map (left). **(G)** Example 2D class averages before (left) and after (right) subtraction of the dominating membrane signal. **(H)** Cryo-EM processing pipeline for CdvB2-coated LNTs in dataset II. Relion jobs are shown in red, CryoSPARC jobs in blue.

Table S1: X-ray Crystallography data collection and refinement statistics

	***S. islandicus* CdvA$^{\Delta C}$** (PDB 9S9G)	***S. islandicus* CdvA$^{\Delta CnoF}$** (PDB 9S9I)	***S. islandicus* CdvB$^{\Delta C}$ (I69M I125M) closed** (PDB 9S9J)	***S. islandicus* CdvB$^{\Delta C}$ (I69M I125M) semi open** (PDB 9S9K)
**Data collection and processing**				
Space group	$P2_1$	C2	$P\ 4_1\ 2_1\ 2$	$P\ 2_1\ 2_1\ 2_1$
Wavelength (Å)	0.97953	1.54179	0.97942	0.97942
Beamline	I03, Diamond Light Source	In-house	I04, Diamond Light Source	I04, Diamond Light Source
a, *b*, *c* (Å)<br>α, β, γ (°)	71.03, 102.72, 328.55, 90.00, 92.35, 90.00	66.33, 78.68, 93.28, 90.00, 104.63, 90.00	59.35, 59.35, 124.37, 90.00, 90.00, 90.00	54.72, 73.34, 97.68, 90.00, 90.00, 90.00
Resolution (Å)	2.9	2.2	2.7	2.2
R_{meas} / R_{pim}	1.711 / 0.358	0.077 (0.864)	0.051 (1.129)	0.093 (0.890)
CC1/2	0.672 (0.137)	0.998 (0.727)	1.0 (0.829)	0.999 (0.779)
I/σ(I)	1.5 (0.2)	9.7 (1.5)	23.8 (2.4)	11.0 (1.9)
Completeness (%)	98.5	96.5 (91.5)	100 (100)	99.9 (99.9)
Multiplicity	26.2	3.0	12.4	6.5 (6.8)
**Refinement**				
Phasing	Molecular replacement: PDB 1PM3	Molecular replacement: PDB 9S9G (this work)	SeMet SAD phasing	SeMet SAD phasing
Molecules per ASU	14	2	1	2
Number of reflections	36,663	22,832	6,575	20,539
R_{work} / R_{free}	0.226 / 0.298	0.228 / 0.259	0.243 / 0.266	0.223 / 0.277
Model composition				
Non-hydrogen atoms	21,978	3,188	1,352	2,856
Protein residues	2,711	384	171	352
Ligands	0	0	0	0
B factors ($Å^2$)				
Protein	68.90	63.43	101.15	57.15
R.m.s. deviations				
Bond lengths (Å)	0.011	0.010	0.007	0.004
Bond angles (°)	1.432	1.353	0.950	0.893
Validation				
MolProbity score	2.2	1.64	1.99	1.78
Clashscore	16.66	13.43	15.68	7.68
Poor rotamers (%)	4.29	0.28	0.00	3.00
Ramachandran plot				
Favoured (%)	98.4	98.15	95.81	98.55
Disallowed (%)	0.04	0.00	0.00	0.00

Statistics for the highest-resolution shell are shown in parentheses.

Table S2: Cryo-EM data collection, refinement and validation statistics

	***S. islandicus* CdvA$^{\Delta C}$** (EMD-54678) (PDB 9S9H)	***S. acidocaldarius* CdvB2 low-twist** (EMD-54673) (PDB 9S97)	***S. acidocaldarius* CdvB2 high-twist class A** (EMDB-54674) (PDB 9S98)	***S. acidocaldarius* CdvB2 high-twist class B** (EMDB-54675) (PDB 9S99)
**Data collection and processing**				
Magnification	75,000	96,000	96,000	96,000
Voltage (kV)	300	300	300	300
Electron exposure (e–/Å^2)	40	35.86	35.86	35.86
Defocus range (μm)	-0.7 to -3.5	-1.0 to -2.6	-1.0 to -2.6	-1.0 to -2.6
Pixel size (Å)	1.068	0.824	0.824	0.824
Symmetry imposed	D1, 48.92° twist, -63.70 Å rise	C2, 1.68° twist, 38.14 Å rise	176.24° twist, 18.82 Å rise	176.92° twist, 18.92 Å rise
Initial particle images (no.)	290,032	580,965	580,965	580,965
Final particle images (no.)	253,803	136,416	139,884	18,345
Map resolution (Å)	4.1	3.2	3.9	4.0
FSC threshold	0.143	0.143	0.143	0.143
**Refinement**				
Initial model used (PDB code)	9S9G (this work)	*De novo* (with ModelAngelo)	*De novo* (with ModelAngelo)	*De novo* (with ModelAngelo)
Map sharpening *B* factor (Å^2)	-139	-83	-125	-90
Model composition				
Non-hydrogen atoms	3130	4549	4530	4728
Protein residues	386	571	567	592
Ligands	0	0	0	0
B factors (Å^2)				
Protein				
R.m.s. deviations				
Bond lengths (Å)	0.005	0.004	0.005	0.005
Bond angles (°)	0.618	0.896	1.05	0.988
Validation				
MolProbity score	1.93	1.28	1.27	1.42
Clashscore	15.25	5.17	5.08	6.65
Poor rotamers (%)	0.57	0.20	0.00	0.19
Ramachandran plot				
Favored (%)	96.34	99.82	99.11	97.77
Allowed (%)	3.66	0.18	0.89	2.23
Disallowed (%)	0.00	0.00	0.00	0.00

Table S3: Structures summary table

<table>
<tr><th></th><th colspan="2">*S. islandicus* CdvA$^{\Delta C}$</th><th>*S. islandicus* CdvA$^{\Delta CnoF}$</th><th colspan="2">*S. islandicus* CdvB$^{\Delta C\ I69M\ I125M}$</th><th colspan="3">*S. acidocaldarius* CdvB2</th></tr>
<tr><td>**NCBI Protein ID**</td><td colspan="2">AGJ62618.1</td><td>AGJ62618.1</td><td colspan="2">WP_012711316.1</td><td colspan="3">WP_011278248.1</td></tr>
<tr><td>**Sequence used in experiment**

mutations are underlined in bold, truncated regions are in grey italics</td><td colspan="2">MPVSYEVLTKFIGQKVKDIYGREFGYLIHVYSEIDGSITGIEVAQGSSILTMGPERIKLDGDSILILPDWKAEAIRILSLMEKIRKRQRALEELYNKQEIPKSDYDDMKRKLDTEMLKVKDDQNKLKGKLKSRLNDIEDQLAHIDKAVISLKMSYISSEIPENAYKGSMEVLRQSKDSYTLERDDIRKTLDRLDSLDKESIELKP*LGSLSTSQQGEAKSDQSKSEIPLPIPVKVINTL*</td><td>MPVSYEVLTKFIGQKVKDIYGREFGYLIHVYSEIDGSITGIEVAQGSSILTMGPERIKLDGDSILILPDWKAEAIRILSLMEKIRKRQR**D**LEE**D**YNKQE**D**PKSDYDDMKRKLDTEMLKVKDDQNKLKGKLKSRLNDIEDQLAHIDKAV**D**SLK**D**SY**D**SSEIPENAYKGSMEVLRQSKDSYTLERDDIRKTLDRLDSLDKESIELKP*LGSLSTSQQGEAKSDQSKSEIPLPIPVKVINTL*</td><td colspan="2">MFDKLPFIFNNEKRRKAQLGKILTEISLKLKDQQTRLEEAIRRLKDRDKELFEKVVRAQVEGDDAKAK**M**YAQEIADIRRIIKVIYTAFLAIEKVRLKLDTVQELQGVSLVLYPVAKILGDLKDQ**M**KGIAPEVAIALDSIISSVNGIAVETGAINDRGVVPAVVDEQARQILDEAQKMAEVKVRELLPDLPHPP*IEQSSRVSQSRPAVRKITERELLDYIVNNGGFLDIEHFSKVYGVEKQEVVKLLEVLKSKGLIAVES*</td><td colspan="3">MADVNDFLRNWGGRQEPTISEKIKNLFKSQQPLRYRLVMANYRLRTTISRLDVYISKLQERDRSLFEKVVESQISKDSARAAMYANEIAEIRKITKQLLTTEIALEQVQLRLETITEIGDIFTSLVPVIGVIRELRNVMKGVMPELSIELADLEEGLQEVVLEAGEFTGARVDFATSSPEARKILDEASAVAEQRMKEKFPSLPSFATSVDQKTNANQK</td></tr>
<tr><td>**Method**</td><td>X-ray crystallography</td><td>Cryo-EM</td><td>X-ray crystallography</td><td colspan="2">X-ray crystallography</td><td colspan="3">Cryo-EM</td></tr>
<tr><td>**Resolution (Å)**</td><td>2.9</td><td>4.1</td><td>2.2</td><td>Closed form: 2.7</td><td>Semi-open form: 2.2</td><td>Low-twist: 3.2</td><td>High-twist class A: 3.9</td><td>High-twist class B: 4.0</td></tr>
<tr><td>**PDB ID**</td><td>9S9G</td><td>9S9H</td><td>9S9I</td><td>9S9J</td><td>9S9K</td><td>9S97</td><td>9S98</td><td>9S99</td></tr>
</table>

Table S4: List of protein expression plasmids used

Plasmid	Description	Source
pOPINS SiCdvA	Untagged full-length CdvA from *S. islandicus*.	This work
pOPINS SiCdvA$^{\Delta C}$	Untagged truncated CdvA from *S. islandicus*, residues 1-205.	This work
pOPINS SiCdvA^{noF}	Truncated CdvA from *S. islandicus*, residues 1-205, with a C-terminal 7His tag and six mutations that prevent polymerisation (A90D, L94D, I100D, I149D, M153D, I156D).	This work
pOPINS SiCdvB	6His-SUMO-CdvB from *S. islandicus*.	This work
pOPINS SiCdvB$^{\Delta C\ I69M\ I125M}$	Truncated 6His-SUMO-CdvB from *S. islandicus*, residues 1-193 and point mutations I69M, I125M.	This work
pOPINS SiCdvB1$^{1\text{-}253}$	Untagged full-length CdvB1 from *S. islandicus*, residues 1-253. For start codon assignment.	This work
pOPINS SiCdvB1$^{11\text{-}253}$	Untagged truncated CdvB1 from *S. islandicus*, residues 11-253. For start codon assignment.	This work
pOPINS SiCdvB1$^{46\text{-}253}$	Untagged truncated CdvB1 from *S. islandicus*, residues 46-253. For start codon assignment.	This work
pOPINS SiCdvB1^{M46}	6His-SUMO-CdvB1 from *S. islandicus*, using newly assigned start codon.	This work
pOPINS SiCdvB2	6His-SUMO-CdvB2 from *S. islandicus*.	This work
pOPINSb SaciCdvA	Untagged CdvA of *S. acidocaldarius*.	This work
pOPINS SaciCdvB	6His-SUMO-CdvB of *S. acidocaldarius*.	This work
pOPINS SaciCdvB1^{M9}	6His-SUMO-CdvB1 of *S. acidocaldarius*, residues 9-214 (most likely start codon based on the *S. islandicus* assignment).	This work
pOPINS SaciCdvB2	6His-SUMO-CdvB2 of *S. acidocaldarius*.	This work
pOPINS SaciCdvB$^{\Delta 15}$	6His-SUMO-CdvB of S. acidocaldarius, residues 16-261.	This work
pOPINS SaciCdvB1$^{\Delta 19}$	6His-SUMO-CdvB1 of *S. acidocaldarius*, with M9 start codon, residues 20-206 (where M9 is residue 1).	This work
pOPINS SaciCdvB2$^{\Delta 27}$	6His-SUMO-CdvB2 of *S. acidocaldarius*, residues 28-219.	This work

References

1. T. Nierhaus, *et al.*, Bacterial divisome protein FtsA forms curved antiparallel double filaments when binding to FtsN. *Nat Microbiol* **7**, 1686–1701 (2022).

2. A. J. McCoy, *et al.*, Phaser crystallographic software. *J Appl Cryst* **40**, 658–674 (2007).

3. J. Agirre, *et al.*, The CCP4 suite: integrative software for macromolecular crystallography. *Acta Cryst D* **79**, 449–461 (2023).

4. K. Cowtan, The Buccaneer software for automated model building. 1. Tracing protein chains. *Acta Cryst D* **62**, 1002–1011 (2006).

5. D. Turk, MAIN software for density averaging, model building, structure refinement and validation. *Acta Cryst D* **69**, 1342–1357 (2013).

6. D. Liebschner, *et al.*, Macromolecular structure determination using X-rays, neutrons and electrons: recent developments in Phenix. *Acta Cryst D* **75**, 861–877 (2019).

7. P. Skubák, *et al.*, A new MR-SAD algorithm for the automatic building of protein models from low-resolution X-ray data and a poor starting model. *IUCrJ* **5**, 166–171 (2018).

8. S. Q. Zheng, *et al.*, MotionCor2: anisotropic correction of beam-induced motion for improved cryo-electron microscopy. *Nature Methods* **14**, 331–332 (2017).

9. A. Rohou, N. Grigorieff, CTFFIND4: Fast and accurate defocus estimation from electron micrographs. *J. Struct. Biol.* **192**, 216–221 (2015).

10. T. Wagner, *et al.*, SPHIRE-crYOLO is a fast and accurate fully automated particle picker for cryo-EM. *Commun Biol* **2**, 1–13 (2019).

11. T. Wagner, *et al.*, Two particle-picking procedures for filamentous proteins: SPHIRE-crYOLO filament mode and SPHIRE-STRIPER. *Acta Cryst D* **76**, 613–620 (2020).

12. S. He, S. H. W. Scheres, Helical reconstruction in RELION. *Journal of Structural Biology* **198**, 163–176 (2017).

13. D. Kimanius, L. Dong, G. Sharov, T. Nakane, S. H. W. Scheres, New tools for automated cryo-EM single-particle analysis in RELION-4.0. *Biochemical Journal* **478**, 4169–4185 (2021).

14. A. Punjani, J. L. Rubinstein, D. J. Fleet, M. A. Brubaker, cryoSPARC: algorithms for rapid unsupervised cryo-EM structure determination. *Nat Methods* **14**, 290–296 (2017).

15. J. Zivanov, T. Nakane, S. H. W. Scheres, A Bayesian approach to beam-induced motion correction in cryo-EM single-particle analysis. *IUCrJ* **6**, 5–17 (2019).

16. J. Zivanov, T. Nakane, S. H. W. Scheres, Estimation of high-order aberrations and anisotropic magnification from cryo-EM data sets in RELION-3.1. *IUCrJ* **7**, 253–267 (2020).

17. D. P. Souza, *et al.*, Evolutionarily conserved principles of ESCRT-III-mediated membrane remodelling revealed by a two-subunit Asgard archaeal system. [Preprint] (2024). Available at: https://www.biorxiv.org/content/10.1101/2024.07.01.601590v1.

18. P. Chai, Q. Rao, K. Zhang, Multi-curve fitting and tubulin-lattice signal removal for structure determination of large microtubule-based motors. *Journal of Structural Biology* **214**, 107897 (2022).
